# Regulation of feather length: FGF/IGF signaling and NOTCH/YAP modulation of progenitor cell topology

**DOI:** 10.1126/sciadv.adw2382

**Published:** 2025-08-22

**Authors:** Ping Wu, Federico Bocci, Christian F. Guerrero-Juarez, Chih-Kuan Chen, George Wang, Tzu-Yu Liu, Jiayi Lu, Shu-Man Hsieh Li, Yung-Chih Lai, Ting-Xin Jiang, Randall B. Widelitz, Ming-Xing Lei, Arthur D. Lander, Qing Nie, Cheng-Ming Chuong

**Affiliations:** ^1^ Department of Pathology, Keck School of Medicine, University of Southern California, Los Angeles, CA 90033, USA.; ^2^ Department of Mathematics, University of California, Irvine, CA 92697, USA.; ^3^ The iEGG and Animal Biotechnology Center, National Chung Hsing University, Taichung, Taiwan.; ^4^ Department of Life Sciences, National Cheng Kung University, Tainan, Taiwan.; ^5^ Center for Craniofacial Molecular Biology, Ostrow School of Dentistry, University of Southern California, Los Angeles, CA 90033, USA.; ^6^ Institute of Biochemistry, National Defense Medical Center, Taipei 114, Taiwan.; ^7^ Integrative Stem Cell Center, China Medical University Hospital, China Medical University, Taichung 40402, Taiwan.; ^8^ Key Laboratory of Biological Science and Technology of Ministry of Education, College of Bioengineering, Chongqing University, Chongqing 400044, China.; ^9^ Department of Developmental and Cell Biology, University of California, Irvine, CA 92617, USA.

## Abstract

The regulation of organ size is a fundamental biological question. This study investigates how feather length is regulated in chickens. We found that collar bulge stem cell zones vary in size: main sickle > lesser sickle > contour feathers. During growth, *IGF* and *FGF9* signaling are highly expressed, while *BMP*, *WIF1*, and *FGF18* increase toward growth termination. Functional assays show that insulin-like growth factor/fibroblast growth factor signaling promotes feather elongation via tyrosine kinase receptor signaling. Single-cell RNA sequencing analysis reveals accelerated differentiation of keratinocytes in short contour feathers compared to long sickle feathers. In Phoenix chickens, superlong main sickle feathers exhibit specialized stem cell zones with enhanced *DLL1* expression and expanded intermediate-layer cell clusters with dynamic interactions involving *NOTCH1*/*DLL1*, *YAP1*, and WNT signaling in progenitor zones in the proximal follicle. Perturbation experiments induce short feather phenotypes arrested at various stages, shedding light on versatile regulatory mechanisms and paving the way for functional control of diverse feather lengths.

## Teaser

FGF/IGF and NOTCH/YAP signaling regulate feather progenitor cells to control feather length.

## INTRODUCTION

Organ size is regulated by both intrinsic and extrinsic mechanisms (*1*–*4*). A key aspect of this process is that organs can sense their size as they grow and adjust accordingly through cell growth and cell cycle progression (*5*). Intrinsic mechanisms involve genetic and epigenetic control over the synthesis and secretion of growth factors, which regulate cell proliferation and differentiation. Extrinsic factors, such as hormones (*5*, *6*), diet (*7*), and mechanical forces (*8*), also play a role in modulating organ size. Together, these factors shape organs during both development and regeneration. While pathways such as insulin-like growth factor (IGF) (*9*) and Hippo (*10*) have been shown to be involved in regulating organ size, much remains to be understood about how molecular signaling integrates with cellular interactions to achieve specific organ dimensions.

In mammalian hair, length is regulated by interactions among Wnt, IGF, bone morphogenetic protein (BMP), and fibroblast growth factor (FGF) pathways (*11*–*14*). These results show that Wnt and IGF signaling are active regulators for hair growth and BMP signaling acts as a negative regulator. FGF pathway members may act in different roles. For example, *FGF7* was demonstrated to effectively stimulate hair germ cells to proliferate and initiate a hair cycle (*15*). *FGF5* regulates hair length by modulating the anagen/telogen transition, and mutations in FGF5 lead to abnormally long body hairs in humans and mice (*16*, *17*).

Feathers evolved independently (*18*) and exhibit different lengths across a bird’s body surface, thus offering a valuable model to study size regulation (*19*). Avian feathers are complex skin appendages with diverse roles in insulation, communication, and flight (*20*). To fulfill these functions, feather length varies, adjusting over a bird’s lifetime in response to both intrinsic growth factors and extrinsic factors such as sex and season (*21*). Thus, they provide a valuable model to study the determinants of organ size.

**Fig. 1.**
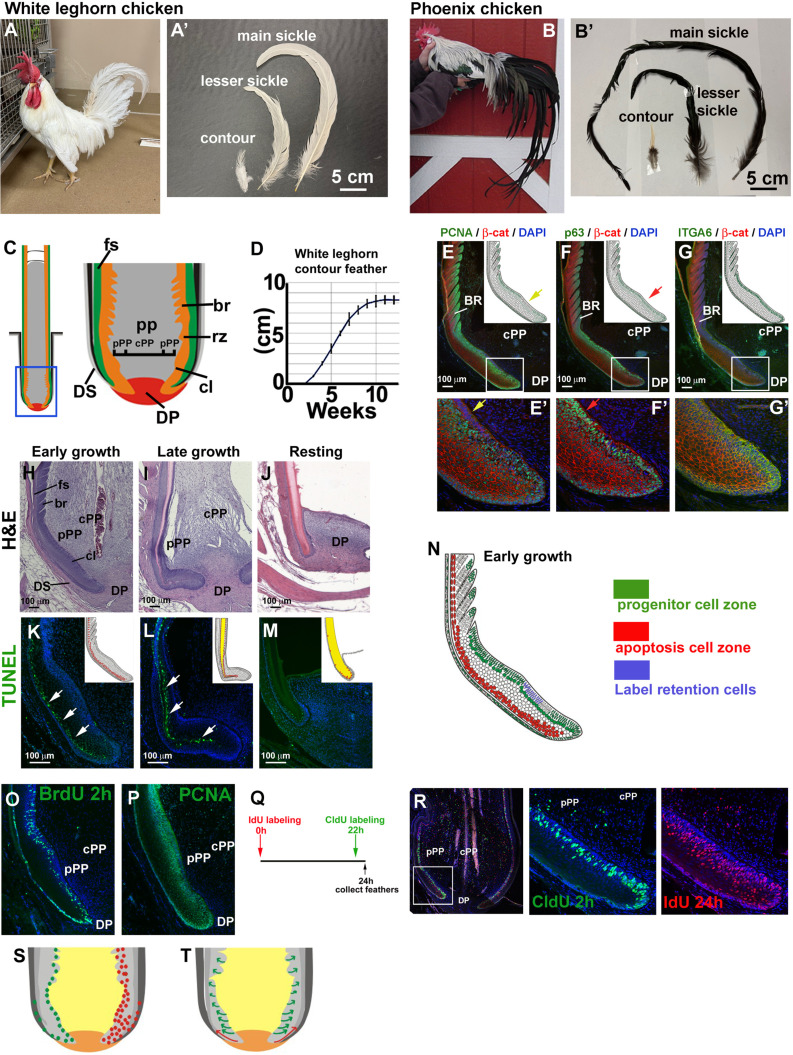
Feather follicle epidermal collar region includes progenitor cell, apoptotic, and differentiation cell zones. (**A**) Adult male White Leghorn chicken. (**A′**) Contour, lesser sickle, and main sickle feathers. (**B**) Adult male Phoenix chicken. (**B′**) Contour, lesser sickle, and main sickle feathers. Note the superlong main sickle feathers in Phoenix chicken. (**C**) Schematic drawing of a growth phase feather follicle. (**D**) Measuring length of White Leghorn chicken regenerating contour feathers. (**E** to **G′**) Immunostaining of an early growth phase contour feather follicle. [(E) and (E′)] PCNA; [(F) and (F′)] p63; [(G) and (G′)] ITGA6. All samples are costained with β-catenin (β-cat; red color). Yellow arrow in (E′) indicates the region negative of PCNA staining, which is equivalent to collar bulge stem cells. Red arrow in (F′) indicates the positive p63 staining region compared to (E′). Insets in (E) to (G) summarize the staining. (**H** to **J**) H&E staining of different stage contour feather follicles. (**K** to **M**) TUNEL staining of early growth, late growth, and resting phase contour feather follicles. White arrows indicate the apoptosis zone. Yellow color in epidermis indicates the keratinized epidermal structure. (**N**) Summary of progenitor cell zone, collar bulge stem cell zone, and apoptosis cell zone in a feather follicle. (**O** and **P**) Two–hour (h) pulse BrdU staining (O) shows narrower progenitor cell zone than PCNA staining (P). (**Q**) Strategy of double TA cell labeling. (**R**) Double TA cell labeling shows the 24-hour pulse has a wider band of positive cells (IdU, red color) than 2-hour pulse (CldU, green color). Middle: Two-hour CldU pulse. Right: Twenty-four–hour IdU pulse. (**S**) Schematic drawing summarizing the double TA labeling pattern. (**T**) The cell migration route revealed by the double TA cell labeling experiment. br, barb ridge; cl, collar; fs, follicle sheath; rz, ramogenic zone.

In this study, we focus on the intrinsic regulation of feather length in dorsal contour and sickle feathers. In adult chickens, dorsal feathers gradually increase in length along the back (*22*). In adult male White Leghorn chickens (Fig. 1A and fig. S1), dorsal contour feathers average around 8 cm, whereas sickle feathers—long feathers located on the posterior back, just before the tail feathers—range from 10 to 36 cm, with the two longest, central feathers referred to as the main sickle feathers (Fig. 1A′). After molting or plucking, feathers at different locations can regenerate to their original length, suggesting that these follicles have a spatial “memory” that modulates molecular activity and the ratio of follicle components, thereby orchestrating feather length in a body position–specific manner. Some chicken breeds, such as the Phoenix chicken, have superlong main sickle feathers (Fig. 1, B and B′) and offer an opportunity to gain further insights into the regulation of feather length.

Measurements of feather length over time reveal that feather growth is determined by two key parameters: growth rate, represented by the slope of the growth curve, and growth period, defined by the duration before growth plateaus. The elongation of keratinocytes may also play a role in feather elongation, but, here, we will focus on the homeotic control of keratinocyte cell numbers. During the growth phase, the feather follicle consists of epithelial and mesenchymal components. The epithelial portion includes the collar, where feather progenitor cells proliferate, and the ramogenic zone, where branching differentiation occurs. Within the collar region, the collar bulge harbors epidermal stem cells, which generate transient-amplifying (TA) cells and differentiated cells during the growth phase (*23*). Over time, the epithelial components of the feather undergo progressive changes, with cells moving from the collar bulge toward the ramogenic zone along the proximal-distal axis (*24*). Eventually, the collar’s TA cells are depleted, marking the transition to the resting phase. At this point, epidermal stem cells descend to the papilla ectodermal region, where they remain until the next feather growth cycle begins (*23*, *25*). We hypothesize that this cyclic control relies on a balance of stem cells, TA cells, and differentiated cells, with dynamic shifts in growth factor signaling influencing the maturation pace of TA cells into differentiated cells. Furthermore, inhibitors may emerge at specific times to signal the end of the growth phase. We propose that the differing growth periods and proliferation rates in short and long feathers are governed by distinct homeostasis mechanisms of stem cells, TA cells, and differentiated cells, all critical to maintaining appropriate organ size (*26*).

In the present study, we investigated the molecular markers and cell clusters in feather collar regions using bulk RNA sequencing (RNA-seq) and single-cell RNA-seq (scRNA-seq). We performed a spatial localization study to identify the distribution of the cell clusters in feathers of different lengths. We found that feather length regulation is dynamic, differing in molecular interactions among cell clusters and in the time required for differentiation. Using replication-competent avian sarcoma virus (RCAS)–mediated functional perturbations, we identified two main strategies used to increase feather length: IGF/FGF function to increase the growth rate and Yes-associated protein (YAP)/NOTCH act to prolong the growth period.

## RESULTS

### Characterization of epidermal progenitor zones in feather collar regions

Normal feather growth includes initiation, growth, and resting phases (*24*, *25*). A White Leghorn chicken contour feather grows for around 9 weeks before entering the resting phase and becoming a fully keratinized mature feather. The architecture of a growth phase feather follicle includes the epidermal and dermal components (Fig. 1C). The epidermal component will develop and keratinize, generating the mature feather structure. The dermal components include the dermal papilla (DP), pulp (PP), and dermal sheath (DS). The peripheral PP (pPP) and feather epidermis interact directly, and vasculature within the central PP (cPP) supplies nutrients.

We wondered whether the feathers grow at a constant rate during their growth phase. To test this, we plucked resting phase contour feathers and measured feather length weekly. We observed that contour feathers grow at a nearly constant rate during the first 7 weeks and then slow down for 2 weeks until they reach the resting phase (Fig. 1D).

To examine feather follicle epidermal cell behavior, we detected progenitor cells within early growth phase contour feathers by immunostaining (Fig. 1, E to G). Both proliferating cell nuclear antigen (PCNA) and p63 staining showed that the feather epidermis cell proliferation zone is adjacent to the pPP (Fig. 1, E, E′, 1F, and 1F′). However, we observed different expression patterns in the feather bulge region where PCNA is negative (Fig. 1E′, yellow arrow) but p63 is positive (Fig. 1F′, red arrow). The feather bulge contains label retention cells (LRCs) that are slow-cycling feather epidermal stem cells (*23*). The distribution of integrin α6 (ITGA6) resembles p63 but exclusively shows membranous staining (Fig. 1, G and G′). Thus, we found a PCNA/p63/ITGA6-positive epidermal cell progenitor zone in feather follicles.

Next, we examined cell apoptosis in early growth, late growth, and resting phase feather follicles. Hematoxylin and eosin (H&E) staining shows their structural differences at these stages (Fig. 1, H to J). At resting phase, the feather epidermis is a fully keratinized structure surrounding the DP (Fig. 1J). Terminal deoxynucleotidyl transferase deoxyuridine triphosphate nick end labeling (TUNEL) staining shows the apoptosis zone in the outer feather epidermis layer (Fig. 1K, white arrows). This apoptosis zone remains in the late growth phase (Fig. 1L, white arrows). Few TUNEL-positive cells are detected at the resting phase (Fig. 1M). PCNA/p63/ITGA6 immunostaining and TUNEL assay data demonstrate that feather follicle epidermis contains a progenitor cell zone and an apoptosis cell zone (Fig. 1N).

To study cell proliferation in the feather follicle, we used a pulsed 5-bromo-2′-deoxyuridine (BrdU) labeling method. We noticed that staining of cells labeled with BrdU for 2 hours shows a narrower progenitor cell zone (Fig. 1O) compared to PCNA staining (Fig. 1P). We wondered whether these patterns might reflect cell migration, since PCNA half-life is greater than 20 hours and may be detected in cells that have finished proliferating. To examine cell migration behavior in the feather epidermis, we designed a two–time point TA cell labeling strategy. To do this, we labeled chickens with 5-Iodo-2′-deoxyuridine (IdU) and 5-chloro-2′-deoxyuridine (CldU) at 0 and 22 hours, respectively, and collected feathers at 24 hours. Thus, feathers were pulse-labeled with CldU for 2 hours and IdU for 24 hours, respectively (Fig. 1Q). CldU (green) and IdU (red) were detected with different specific antibodies. As expected, we found markedly different staining patterns between cells labeled for 2 or 24 hours. Twenty-four–hour labeling produced a much wider cell band than the 2-hour labeling (Fig. 1R). An enlarged view displays the possible cell movement route in Fig. 1R (middle and right). This double TA cell labeling result (Fig. 1S) reveals two migration routes. One route shows progenitor cells close to the DP moving upward toward the apoptosis zone (Fig. 1T, red arrows). The second route suggests that progenitor cells above the DP move outward and upward to form the feather branch (Fig. 1T, green arrows).

These results demonstrate that the feather collar epidermis includes distinct progenitor, migration, and apoptosis zones. The progenitor cell zone and migration zone may provide the cells for feather branching; the latter will undergo terminal differentiation and become the final feather structures. The apoptosis zone may contribute to forming the feather sheath.

### Comparing LRC/TA activities in feathers with different lengths

Adult birds have feathers with different lengths across their body. Male White Leghorn chickens have their longest main sickle feather in the center and five shorter lesser sickle feathers that taper in length on each side. On their dorsal tract, they have shorter contour feathers. Here, we compare these three types of chicken feathers, e.g., contour, lesser sickle (no. 3), and main sickle feathers (Fig. 1A′) to explore factors regulating feather growth. The average length of contour feathers is 8.3 cm, whereas main sickle feathers can grow to 36 cm long.

**Fig. 2.**
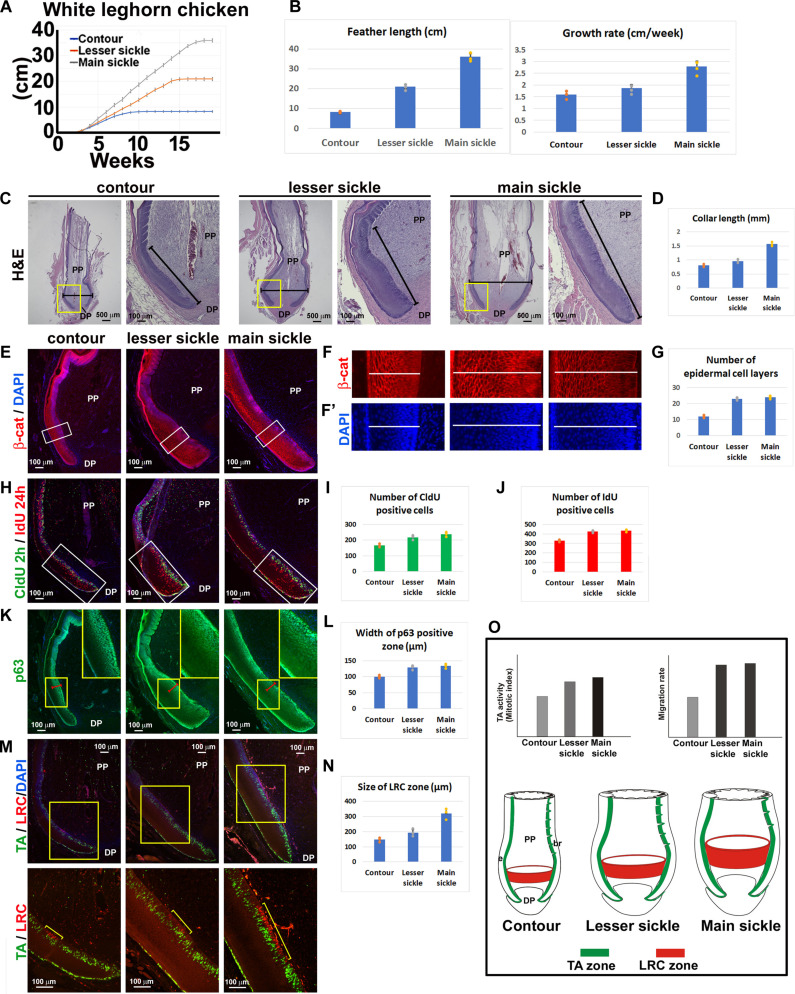
Comparing the collar regions of contour, lesser sickle, and main sickle feather follicles in White Leghorn chickens. (**A**) Differences of growth rate and growth period in regenerating contour, lesser sickle, and main sickle feathers of White Leghorn chicken (*n* = 3). (**B**) Comparison of full feather length and growth rate among contour, lesser sickle, and main sickle feathers (*n* = 3). (**C**) H&E staining shows the comparative size and structure of growth phase contour, lesser sickle, and main sickle feathers. The horizontal lines are used for follicle width measurements. The tilted lines are used at high magnification for collar length measurements. (**D**) Statistics of growth phase follicle collar length among contour, lesser sickle, and main sickle feathers (*n* = 3). (**E**) Comparison of β-catenin expression among contour, lesser sickle, and main sickle feathers. DAPI is used for counterstaining. (**F** and **F′**) β-Catenin and DAPI staining in higher magnification. The white lines in (F) and (F′) are used to count the epidermal cell layer numbers. (**G**) Statistics of growth phase follicle epidermal cell layer numbers among contour, lesser sickle, and main sickle feathers (*n* = 3). (**H**) Double TA staining in the samples with 2-hour CldU (green) and 24-hour IdU (red) labeling. The white rectangular boxes were used to measure the CldU- and IdU-positive cells. (**I** and **J**) Count of CldU- and IdU-positive cell numbers in the epidermis among contour, lesser sickle, and main sickle feathers (*n* = 3). (**K** and **L**) p63 staining and measurement of the p63-positive zone in the feather epidermis. (**M**) LRC/TA cell double staining. The LRC zone is indicated by the yellow line. (**N**) Size of LRC zone among contour, lesser sickle, and main sickle feathers (*n* = 3). (**O**) Summary of mitotic index, migration rate, and LRC configuration differences in different sized feathers.

We wondered whether different feather lengths are due to the difference in growth rate or growth duration. To answer this question, we plucked three types of feathers to induce a feather cycle and measured the growth rate weekly. We found that main sickle feathers have the fastest growth rate and the longest growth duration, whereas contour feathers have the slowest growth rate and the shortest growth duration (Fig. 2, A and B). Thus, our data suggest that both growth rate and growth duration contribute to the final feather length.

H&E staining from sections of early growth phase follicles shows that the main sickle feathers have the widest follicle size (horizontal line) and longest collar length (tilted line), whereas contour feathers have the narrowest follicle width and shortest collar length (Fig. 2, C and D). These results suggest that longer feathers have more complex follicular architecture to support the higher rate and duration of growth. β-Catenin immunostaining and 4′,6-diamidino-2-phenylindole (DAPI) staining in the collar epidermis show that there are more cell layers in both main and lesser sickle feathers compared to contour feathers (Fig. 2, E to G). We used the double TA-labeling strategy (Fig. 1Q) to examine these three types of feather follicles (Fig. 2H). The main sickle feather epidermis has the highest number, while contour feathers show the lowest number of 2-hour-pulse CldU-positive cells (Fig. 2I). A 24-hour-pulse IdU labeling shows that the epidermises of main sickle and lesser sickle feathers have similar numbers of IdU-positive cells, which are higher than that seen in contour feathers (Fig. 2J). These results suggest that bigger feathers have higher mitotic rates and migration rates, whereas similar width feathers have similar migration rates.

We further examined p63 immunostaining to compare the thickness of the p63-positive zone among these three types of feathers. Both sickle feathers show a wider p63-positive zone than contour feathers (Fig. 2, K and L). Thus, p63 staining suggests that long feathers have wider progenitor cell zones.

Furthermore, we compared the apoptosis zone and epidermal cell differentiation in different feather follicles (fig. S2). We found that the main sickle feathers have the widest apoptosis zone (fig. S2A, blue arrow). We used α-keratin (K17) and β-keratin immunostaining to show the differentiating cells in the feather follicle. In hair, K17 is expressed during the early stages of hair follicle development and in cycling anagen follicles, especially in the progenitor cell–rich regions such as the outer root sheath, matrix, and medulla compartment (*27*–*29*). β-Keratin is specifically expressed in the reptile and bird skin appendages (*30*–*32*). In feather follicles, K17 is expressed not only in the collar progenitor region (red arrows) but also in the intermediate layer (yellow arrows), barb ridge basal layer cells (white arrows), and the feather sheath (pink arrows) (fig. S2, B to D). In contrast, β-keratin is expressed in the feather sheath (white arrowhead) and the differentiating barb ridge (red arrowhead) (fig. S2, E to G). The main sickle feather only showed the barb ridge β-keratin expression in the more distal epidermis (fig. S2G, yellow arrowhead), suggesting the expansion of the progenitor cell pool and delay of keratin differentiation in longer feather follicles (fig. S2H).

We next examined the LRCs in these three feather follicles (Fig. 2M). LRCs are feather stem cells that locate in the collar region (collar bulge) (*23*). We found that the LRC zone size correlates with feather length, with the biggest LRC zone in main sickle feathers and the smallest in contour feathers (Fig. 2, M and N). Thus, we conclude that longer feathers have a higher mitotic rate, higher migration rate, wider progenitor cell zone, and bigger LRC zone (Fig. 2O).

### Bulk transcriptomics reveals dynamic molecular profiles during feather cycling

**Fig. 3.**
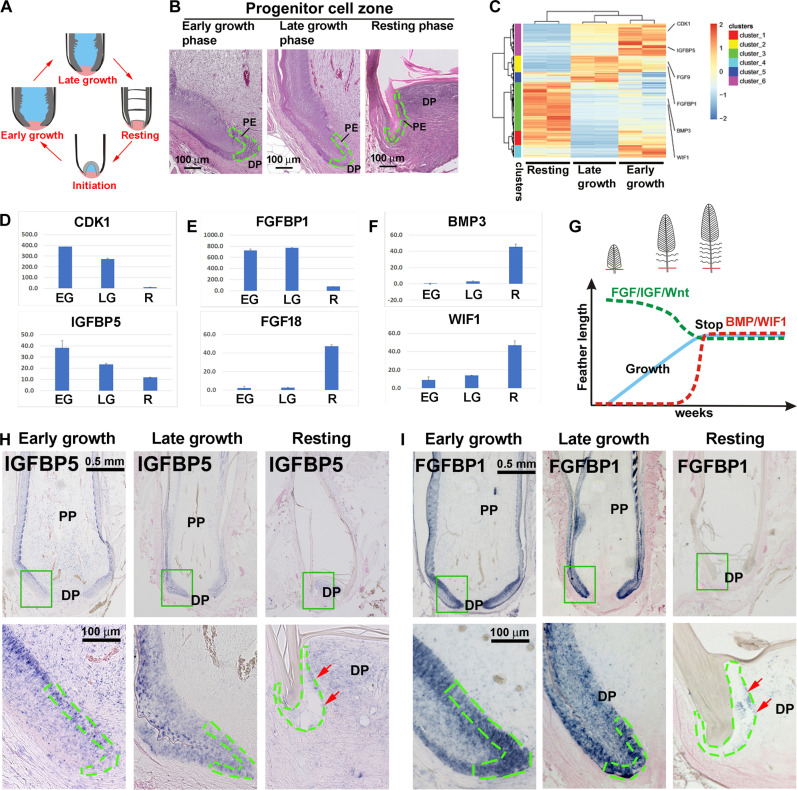
RNA-seq of epidermal progenitor cell zone reveals the molecular circuit regulating feather cycling. (**A**) Schematic drawing shows feather cycling. (**B**) Epidermal progenitor cell zone dissection from early growth (EG), late growth (LG), and resting (R) phase feather follicles. Green dashed lines indicate the papilla ectoderm (PE) surround the DP. Papilla ectoderm was dissected, and bulk RNA-seq was performed. (**C**) Hierarchical clustering of the epidermal progenitor cell zone from early growth, late growth, and resting phase feather follicles. Whole feather epidermis is included for comparison purposes. (**D**) Examples of molecules with declining expression level trends from early growth to resting phase. (**E**) Examples of FGF signaling expression trends from early growth to resting phase. (**F**) Examples of molecules with increasing expression level trends from early growth to resting phase. (**G**) Schematic summary of molecular expression in different growth phase epidermal progenitor cells. Progenitor cell activities will be reduced when entering the resting phase. (**H** and **I**) Section in situ hybridization in early growth, late growth, and resting phase feather follicles. (H) *IGFBP5*; (I) *FGFBP1*. Red arrows show the increased expression in resting phase epidermis. Green dashed lines indicate papilla ectoderm.

Comparing different feather types suggests that the progenitor cell configuration is important for size regulation (Fig. 2). A single feather’s life span can go through initiation, early growth, late growth, and resting phases (Fig. 3A). The duration of the growth phase may determine the final feather length. However, molecules controlling the termination of feather growth and entry to the resting phase are unknown. To understand the putative molecular circuit regulating feather epidermal cycling, we isolated cells from the progenitor cell zone from different growth phases (early growth phase, late growth phase, and resting phase) and performed bulk RNA-seq (Fig. 3, B and C).

Analysis reveals 2057 differentially expressed genes (DEGs) between epidermal progenitor cells in early growth and late growth phases. Among them, 1462 genes are up-regulated in early growth phase, whereas 595 genes are up-regulated in late growth phase. At the transition from late growth phase to resting phase, we found 3921 DEGs. Among them, 1505 genes are up-regulated in late growth, whereas 2416 genes are up-regulated in resting phase (table S1). Gene ontology analyses from early growth phase versus late growth phase and late growth phase versus resting phase are shown in table S2. Protein binding was shown as the most enriched molecular function and system development as the most enriched biological process.

Examples of molecules showing declining, increasing, and then declining or increasing expression level trends among different regeneration stages are shown in Fig. 3 (D to F), respectively. Among the molecules with declining trends, *CDK1* is related to cell proliferation. The IGF pathway (*IGFBP5*) also shows declining expression. Signaling molecules including the FGF pathway (*FGFBP1* and *FGF18*) show different trends. Among molecules with the increasing trend, the BMP pathway (*BMP3*) and a WNT pathway antagonist (*WIF1*) are shown in Fig. 3F. This result suggests that BMP and WNT signaling pathways are involved in regulating feather epidermal progenitor cell proliferation and migration. Reducing progenitor cell activity is necessary to end the growth phase, thus determining the length of feathers (Fig. 3G).

The heatmap of FGF and IGF pathway members are further highlighted in fig. S3. FGF pathway members show different trends. *FGF6*, *FGF9*, *FGFBP1*, and *FGFR1* are increased in the growth phase, whereas *FGF1*, *FGF2*, *FGF18*, *FGF23*, *FGFR2*, and *FGFR3* are increased toward the end of growth phase, implying more complex roles of FGF signaling in feather length control.

Examples of in situ hybridization of *IGFBP5* and *FGFBP1* are shown in Fig. 3, (H and I), respectively. The expression levels and domains of both molecules become nearly negative in resting phase epidermis (red arrows), which prompts us to ask whether IGF and FGF pathways involved feather size control as activators.

### Single-cell transcriptomics identifies distinct cell clusters in proximal follicles

**Fig. 4.**
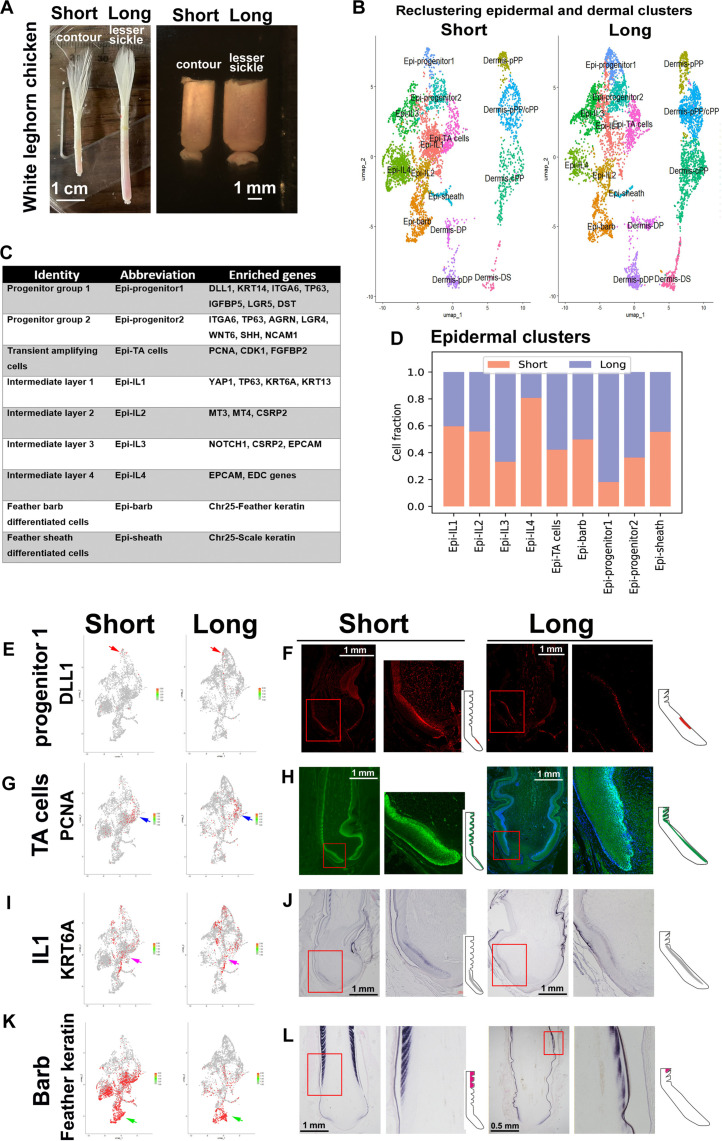
scRNA-seq of contour feathers and lesser sickle feathers from White Leghorn chickens reveals the epidermal cell configuration difference between short and long feathers. (**A**) Contour and lesser sickle feather follicles from White Leghorn chicken are used for scRNA-seq analysis. (**B**) Nine epidermal cell clusters and six dermal clusters are identified in short and long feathers, with different cell numbers in different cell clusters. (**C**) Marker genes in epidermal cell clusters. (**D**) Cell fraction in epidermal clusters between short and long feathers. (**E** to **L**) Examples of the expression of different cell markers in short and long feather epidermal cell clusters. [(E) and (F)] Progenitor cell group 1 (*DLL1*). [(G) and (H)] TA cells (PCNA). [(I) and (J)] Intermediate layer 1 (IL1) and keratin 6A (*KRT6A*). [(K) and (L)] Barb-differentiated cell cluster (*feather keratin*). Red arrows in (E) indicate the progenitor cell group 1. Blue arrows in (G) indicate the TA cells. Pink arrows in (I) indicate the intermediate layer 1. Green arrows in (K) indicate the differentiated cell cluster.

To further understand cellular activity among different sized feather follicles, we performed scRNA-seq for regenerating White Leghorn chicken contour feathers and lesser sickle feathers (Fig. 4A and fig. S4A). We compare two feather types, contour and lesser sickle, as representatives for short and long feathers. We identified a total of 30 cell clusters, with 15 clusters belonging to epidermal and dermal groups (fig. S4B and table S3). Upon further reclustering, eight epidermal and six dermal clusters emerged (Fig. 4B and table S4). Dermal cell clusters include specific groups such as pPP, cPP, pPP/cPP (PP cells expressing both pPP and cPP markers), DP, pDP (peripheral DP), and DS.

We focus here on epidermal cell clusters. In total, 3752 epidermal cells from short feathers and 3559 from long feathers were analyzed. Cluster 3 is of particular interest, as it expresses markers of epidermal progenitor cells, including *KRT5*, *ITGA6*, and *Lgr5*. Further subclustering of this group revealed two distinct progenitor subgroups (table S5). Together, the feather epidermis is composed of two progenitor cell clusters, one TA cell cluster, four intermediate layer clusters, and two terminally differentiated clusters (feather barb and feather sheath), with each cluster exhibiting unique gene expression profiles (Fig. 4C). Progenitor cells proliferate, migrate, and eventually some differentiate into feather sheath or barb cells (Fig. 1T). Some later will undergo apoptosis (Fig. 1N).

To check the consistency and reproducibility of feather follicle scRNA-seq, we compared the data from two short feather follicles (fig. S5). The two samples show the same cell clusters and similar cell fraction ratios (fig. S5, A and B). The expressions of major differential markers are also similar (fig. S5C). These data prove the reproducibility of our scRNA-seq analysis.

### Single-cell transcriptomics analyses for short and long feathers

When comparing epidermal cell clusters between short and long feathers, we observed a notable difference in cell distribution using scRNA-seq data. Long feathers exhibited a greater number of cells in progenitor cell groups 1 and 2, as compared to short feathers (Fig. 4D). To further explore these findings, we used immunostaining, RNAscope, and in situ hybridization techniques to localize each cell cluster within the feather follicles (fig. S6). We observed that progenitor cell group 1, TA cells, intermediate layer 1, and terminal differentiated barb cell clusters were organized within the feather space (Fig. 4, E to L).

Long feathers show higher expression of the progenitor cell marker *DLL1* (Fig. 4E, red arrow). This is supported by RNAscope analysis, which revealed an expanded *DLL1* expression zone in long feathers (Fig. 4F). No substantial differences in TA cell numbers were observed between short and long feathers [Fig. 4, G (blue arrow) and H]. In the intermediate layer 1, high expression levels of *KRT6A* were evident, indicating its presence beyond the TA cell zone in both feather types [Fig. 4, I (pink arrow) and J].

To further analyze IGF and FGF signaling, we quantified the strength of IGF and FGF gene signatures in epidermal cells. Our comparative analysis revealed that both IGF and FGF pathways were consistently up-regulated across all epidermal clusters in long feathers, with expression fold changes ranging from 1.5 to 7 (fig. S7, A to D). Notably, the expression of IGF and FGF pathways demonstrated a high correlation in individual cells, even after adjusting for shared genes within both pathways (fig. S7E).

Short feathers are observed to contain more keratin-expressing cell clusters. Feather keratin, a terminal differentiation marker for feather barb ridge cells, displayed the highest expression levels in the barb-differentiated cell cluster of both short and long feathers (Fig. 4K, green arrows). However, three intermediate layer clusters showed low levels of feather keratin expression in short feathers (Fig. 4K, left), which is not observed in the intermediate layer clusters of long feathers (Fig. 4K, right). In situ hybridization revealed that the feather keratin gene expression domain is situated closer to the DP in short feathers (Fig. 4L, left). In addition, we found that scale keratin genes, which are not only found in scales but also present in hard integument (*30*), are expressed within the feather sheath–differentiated cell cluster (fig. S6J). In situ hybridization showed that scale keratin genes are highly expressed in the feather epidermis apoptotic zone, which eventually contributes to feather sheath formation (fig. S6J).

**Fig. 5.**
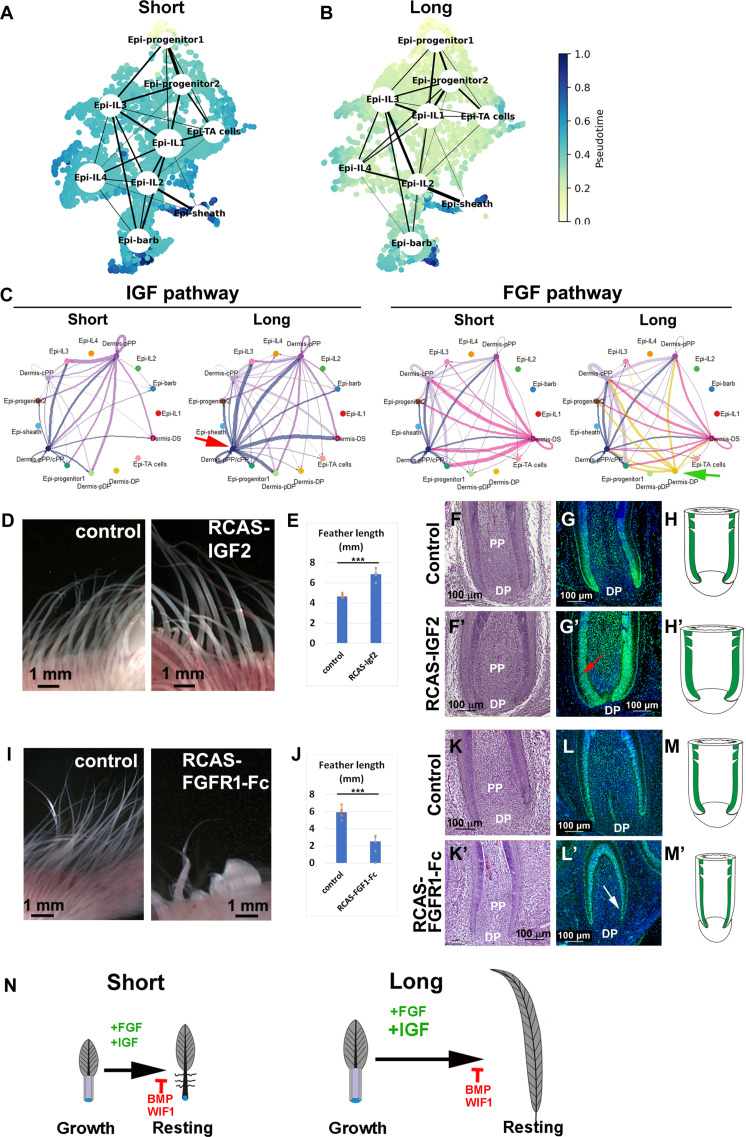
Pseudotime, CellChat, and functional analysis on the growth of short and long feathers. (**A** and **B**) Pseudotime analysis in short and long feathers. Color map indicates diffusion pseudotime or the progression along the epidermal lineage, while the thickness of solid black lines indicates the probability of transition between epidermal cell states. Intermediate layer and TA cells are predicted to transition faster toward the differentiated epidermal states in the short feather, as demonstrated by the higher pseudotime value. (**C**) The comparison of IGF and FGF pathway interaction strength between short and long feathers. Top: IGF pathway. Bottom: FGF pathway. Red arrow indicates the stronger IGF pathway interaction in long feathers. Green arrow indicates a specific FGF pathway interaction in long feathers. (**D** and **E**) Overexpression of IGF2 in the right wing leads to increased feather filament length (E13). (**F** to **H′**) Comparison of H&E and PCNA staining between control and IGF2 overexpressed samples. Note the enlarged feather follicle width (F′) and the expanded PCNA expression domain (G′) (red arrow) in the RCAS-IGF2–injected sample on the right wing, compared to the control follicle in the left wing. (**I** and **J**) Inhibition of FGF signaling by RCAS-FGFR1-Fc can significantly reduce the feather length (E13). (**K** to **M′**) Comparison of H&E and PCNA staining between control and RCAS-FGFR1-Fc–infected samples. Note the reduced feather follicle width (K′) and the reduced number of PCNA-positive cells (L′) (white arrow) in the RCAS-FGFR1-Fc–infected sample from the right wing, compared to the control follicle in the left wing. (**N**) Summary of activated IGF and FGF pathway activities in short and longer feathers. For [(E) and (J)], five flight feather filaments from the right (experiment) and left (control) wings were measured. ***P < 0.001.

To further elucidate epidermal lineage progression, we calculated diffusion pseudotime (*33*, *34*) and assessed the transition probabilities between epidermal cell states in both short and long feathers. Notably, the intermediate layer and TA cells in short feathers were predicted to transition more rapidly toward terminal epidermal states, as indicated by their higher pseudotime coordinates (Fig. 5A). In contrast, progenitor cells in long feathers exhibited slower differentiation, evidenced by the significant discrepancy in pseudotime values between intermediate/TA cells and barb/sheath cells (Fig. 5B). This emerging difference in differentiation speed between short and long feathers is mirrored in the distinct expression patterns of feather keratin. Overall, our pseudotime analysis supports the notion that epidermal cells in long feathers follow a more prolonged pathway to achieve terminal differentiation.

To understand how these differentiation-inducing signals are coordinated in the tissue, we used CellChat (*35*) to assess the interaction strengths between short and long feathers, including nine epidermal clusters and six dermal clusters in our analysis. In general, dermal clusters mainly acted as IGF senders (cPP) or senders/receivers (pPP and pPP/cPP), whereas epidermal clusters (IL3 and progenitor 1) mainly acted as receivers. Our findings, however, indicated that long feathers exhibited stronger IGF pathway interactions between the PP (dermal pPP/cPP) and epidermal clusters (Fig. 5C, red arrow), in good agreement with the stronger intracellular IGF signature previously identified in the epidermal cells from the long feather (fig. S7, A to D). While the overall network topology of IGF-based cell-cell communication was largely conserved between short and long feathers, epi–IL-1 and dermis-DS cells assumed a markedly stronger role as IGF receivers in long feathers by expressing *ITGAV* and *ITGB3*, respectively (fig. S8, A and B). The FGF pathway follows a similar trend, whereby dermis and epithelial clusters act as senders and receivers, respectively. Long feathers displayed a specific interaction between the DP (dermis-DP) and other clusters (Fig. 5C, green arrow) due to long feather–specific expression of the ligands *FGF7* and *FGF10* in dermis-DP (fig. S8, C and D).

To further identify the critical signaling nodes in epidermal cell differentiation, we used spliceJAC (*36*) to reconstruct the gene regulatory networks (GRNs) associated with the IGF/FGF pathways, specifically focusing on the gene centrality (fig. S7, F and G). The gene centrality score measures the connectivity of a given gene with other genes within the GRN, with higher scores indicating greater involvement in gene regulation. Through ranking genes by centrality score, we identified three genes—*SOS1*, *MAPK8*, and *MAPK3*—that exhibited high centrality and are shared by both IGF and FGF signaling pathways (fig. S7, H and I).

Together, the quantification of the IGF/FGF intracellular response, reconstruction of cell-cell communication (CCC) network, and downstream GRN inference support a model, whereby IGF/FGF signaling is intensified in the long feather, specifically activating dermal cell clusters (dermis-DS and dermis-DP) that are inactive in the short feather.

### FGF/IGF pathways regulate feather length by modulating cell proliferation

We observed that the length of developing and regenerating chicken feathers maintains a strict left-right equal growth pattern, e.g., the length of a flight feather from the left wing is always similar to the length of a feather growing from an equivalent position on the right wing. We performed control experiments in which we injected RCAS–green fluorescent protein (GFP) into the right wing at embryonic day 4 (E4). None of those samples showed significant differences in feather length between the left wing and right wing (*n* = 6/6).

To examine the role of the IGF pathway in feather size control, we overexpressed IGF2 in the chicken embryo. RCAS-IGF2 virus was injected to the right-wing bud at E4. Samples were collected at E13, and the length of the feather filament from both the left wing and right wing was measured. The feather filament in the right wing is significantly longer than that in the left wing (Fig. 5, D and E; *n* = 8/8 embryos). Sections of embryonic feather follicles expressing ectopic IGF2 show increased follicle widths (Fig. 5F′ compared to (Fig. 5F) accompanied with an expanded PCNA expression zone (Fig. 5G′ compared to Fig. 5G, red arrow). These results indicate that the IGF signaling increases cell proliferation and expands the TA cell zone (Fig. 5, H′ versus H).

To evaluate the role of the FGF pathway in feather size control, we used a secreted dominant-negative version of FGF receptor 1 (FGFR1) (RCAS-FGFR1-Fc) to suppress FGF signaling in the right-wing bud. In early embryonic feather development, RCAS-FGFR1-Fc could block feather placode initiation (*37*). We found that flight feather follicles still can form but the length of feather filaments is significantly reduced (Fig. 5, I and J; *n* = 6/6 embryos). These feather follicles have a narrower width (Fig. 5K′ compared to Fig. 5K) and reduced PCNA-positive domain in the follicle base (Fig. 5L′ compared to Fig. 5L, white arrow). These results indicate that the FGF signaling is important to maintain TA cell activities and feather elongation (Fig. 5M′ compared to Fig. 5M).

We performed more IGF/FGF pathway functional studies to verify the role of IGF/FGF in the feather size regulation. When using RCAS-dnIGF1R (dominant-negative version of IGF1R), we found that feather length is significantly reduced (fig. S9, A and B; *n* = 5/5 embryos). The follicle width is reduced accompanied by the narrower PCNA expression zone (fig. S9, C to E′). When we used RCAS-FGF8 to increase FGF signaling, the feather filament was shortened, but the width increased significantly (fig. S9, F and G; *n* = 6/6 embryos). PCNA staining shows that there is an ectopic proliferation zone in RCAS-FGF8–treated samples (red arrows; fig. S9, H to J′). Furthermore, we used RCAS-Spry4 to suppress FGF/epidermal growth factor signaling (*38*). The feather filament length is reduced in the treated samples (fig. S9, K and L; *n* = 6/7 embryos). PCNA is negative in the base of treated follicles (yellow arrows; fig. S9, M to O′). By characterizing the perturbed feather follicles, we can appreciate that the length represents a balance between growth and differentiation processes. Shortened feathers can be caused by precocious keratinocyte differentiation or failure of progenitor cells to differentiate.

Together, the quantification of signaling strength, reconstruction of GRN, and the functional studies imply a working model in which IGF and some FGF members are strongly activated during the early differentiation stages of epidermal cells, while BMP, Wnt inhibitory factor 1 (WIF1), and some other FGF members are higher toward the end of growth phase to initiate differentiation (Fig. 5N).

### YAP/NOTCH/DLL regulates feather length by modulating progenitor population size

**Fig. 6.**
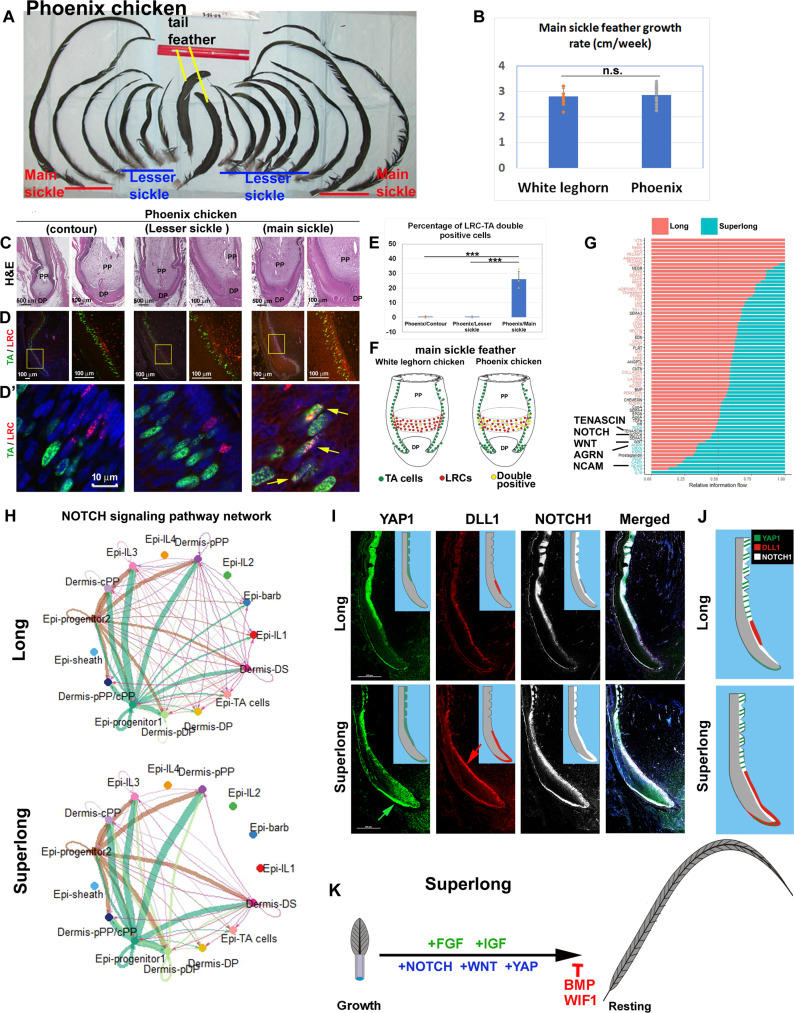
The superlong main sickle feathers in Phoenix chicken exhibit distinct progenitor cell zone and molecule pathways. (**A**) Examples of main sickle, lesser sickle, and tail feathers collected from a 1-year-old male Phoenix chicken. (**B**) Comparison of main sickle feathers between White Leghorn chicken and Phoenix chicken shows similar growth rate. For White Leghorn chicken, *n* = 13. For Phoenix chicken, *n* = 22. n.s., not significant. (**C**) Comparison of regenerated contour, lesser sickle, and main sickle feathers from Phoenix chickens. (**D**) LRC and TA double staining. (**D′**) High magnification. Yellow arrows indicate the LRC/TA–double positive cells. (**E**) Calculation of percentage of LRC/TA–double positive cells. ***P < 0.001. (**F**) Schematic drawing to summarize the epidermal progenitor cell zone configuration difference between main sickle feathers in White Leghorn chickens and Phoenix chickens. (**G**) CellChat analysis shows the increased cell-cell interaction in superlong Phoenix main sickle feathers. (**H**) Comparison of NOTCH pathway cell-cell interactions in White Leghorn chicken long feathers and Phoenix chicken superlong feathers. (**I**) RNAscope analysis of YAP1/DLL1/NOTCH1 showing DLL1 expression domain are expanded (red arrow). In Phoenix chicken superlong feathers, the expression patterns of both NOTCH1 and YAP1 are expanded and curve around the follicle base, extending toward the DS (green arrow). (**J**) Summary of YAP1/DLL1/NOTCH1 expression in White Leghorn chicken and Phoenix chicken sickle feather follicles. (**K**) Summary of enhanced NOTCH, WNT, and HIPPO pathway activities in superlong Phoenix sickle feathers.

Phoenix chicken main sickle feathers can exceed 100 cm in length. The dynamic change of feather follicle configurations seen among different sized feathers prompted us to ask how the extreme long sickle feathers in male Phoenix chickens are generated (Fig. 6A). We wondered whether this is based on the growth rate or growth period. We measured the elongation of contour, lesser sickle, and main sickle feathers weekly. We found that the growth rates of main sickle feathers between White Leghorn and Phoenix chickens are similar (Fig. 6B and fig. S1C). On the other hand, the growth period of the Phoenix chicken main sickle is more than 8 months versus 3 to 4 months in White Leghorn chickens. Thus, growth duration appears to be the determinant that controls the final length of main sickle feathers.

To study Phoenix feather follicles further, we plucked main sickle feathers from the resting phase feathers and collected the regenerating feathers. For comparison, regenerated contour and lesser sickle feathers from Phoenix chicken were also collected. H&E staining shows that, similar to the trend in White Leghorn chickens, Phoenix chicken main sickle feather follicles have the biggest collar width and length (Fig. 6C). PCNA and p63 staining show that the Phoenix chicken main sickle feathers have an enlarged TA cell zone and progenitor zone (fig. S10A). We also noticed that, unlike the contour and lesser sickle feathers that have a PCNA-negative area in the collar bulge area (white arrows in fig. S10A), the Phoenix chicken main sickle feather follicles lack this area (red arrows). TA/LRC double labeling experiments show that the LRCs are mingled with the TA cell zone in Phoenix chicken main sickle feather follicles (Fig. 6D). Confocal imaging shows that few LRCs (IdU-labeled, red color) are double stained with green (TA cells, CldU-labeled) in Phoenix chicken contour and lesser sickle feather follicles, whereas more than 26% of LRCs in Phoenix chicken main sickle feather follicles are double labeled with both red and green [Fig. 6, D′ (yellow arrows) and E]. Thus, the mingled LRC/TA zone in the bulge area suggests that the collar bulge stem cells are less quiescent and may act to support the longer growth of these extremely long feathers (Fig. 6F).

To analyze molecular differences, we collected the regenerating Phoenix chicken main sickle feather follicles and performed scRNA-seq. When we compare the IGF/FGF/WNT pathway, we observed that Phoenix chicken superlong feathers do not exhibit more interactions in IGF and FGF pathways, compared to long White Leghorn feathers (fig. S11, A and B). However, these superlong feathers have more WNT signaling interactions between progenitor 1 and progenitor 2 (red arrow in fig. S11C). This suggests that these superlong feathers use a different mechanism to increase their length. When compared to White Leghorn chicken long feathers, we did not find different cell clusters that existed in Phoenix chicken superlong feathers. However, CellChat analysis (*35*) revealed that the Phoenix superlong feathers exhibit more interactions in several pathways including the NOTCH, WNT pathways, and extracellular matrix (Fig. 6G). The AGRN pathway also shows high activity level in Phoenix chicken superlong feathers. The AGRN pathway is related to YAP signaling, and it was proposed as a mechanotransduction signal regulating YAP through the Hippo pathway (*39*). Examples of enhanced interactions are shown in Fig. 6H and fig. S10 (B and C), including NOTCH, tenascin C (TNC), and neural cell adhesion molecule (NCAM) signaling.

We further localized the expression of candidate genes focusing on *YAP1*, *DLL1*, *NOTCH1*, *TNC*, and *NCAM* in the feather follicle. RNAscope analysis (Fig. 6, I and J) shows that *YAP1*/*DLL1*/*NOTCH1* are expressed in distinct domains in White Leghorn sickle feathers (Fig. 6I, top). *DLL1* is expressed in the collar bulge region (equivalent to the LRC domain). *NOTCH1* is expressed in the intermediate layer close to the progenitor zone and is negative in the *DLL1* expression domain. *YAP1* is expressed above the *DLL1* domain and partially overlaps with the *NOTCH1* expression domain (summarized in Fig. 6J, top). However, The *DLL1* expression zone is greatly expanded in Phoenix chicken superlong feathers (Fig. 6I, red arrow, and table S6). *YAP1* expression is extended and curves around the follicle base to be present in the lower DS (Fig. 6I, green arrow). The expanded *DLL1* and *YAP1* expression domain in Phoenix chicken superlong feather follicles may reflect the altered configuration of progenitor clusters and their interactive interfaces (Fig. 6F). Superlong feathers also show high expression levels of *TNC* and *NCAM* (fig. S10D, green and yellow arrows), implying alterations in cell-substrate and cell-cell interactions.

To test whether the *DLL1* expression domain is restricted in the collar bulge, we costained the *YAP1*/*DLL1* and TA cells in White Leghorn contour feathers (fig. S12). *DLL1* expression was found to be surrounded by TA cells in the collar bulge zone, supporting the idea that *DLL1*-positive cells represent collar bulge stem cells (fig. S12B, yellow arrow). Thus, we found that the superlong Phoenix chicken sickle feathers have specialized progenitor cell topology, expanded progenitor cell populations, and increased YAP/NOTCH/WNT signaling (Fig. 6K).

### Perturbing YAP/NOTCH signaling reveals distinct functional effects

**Fig. 7.**
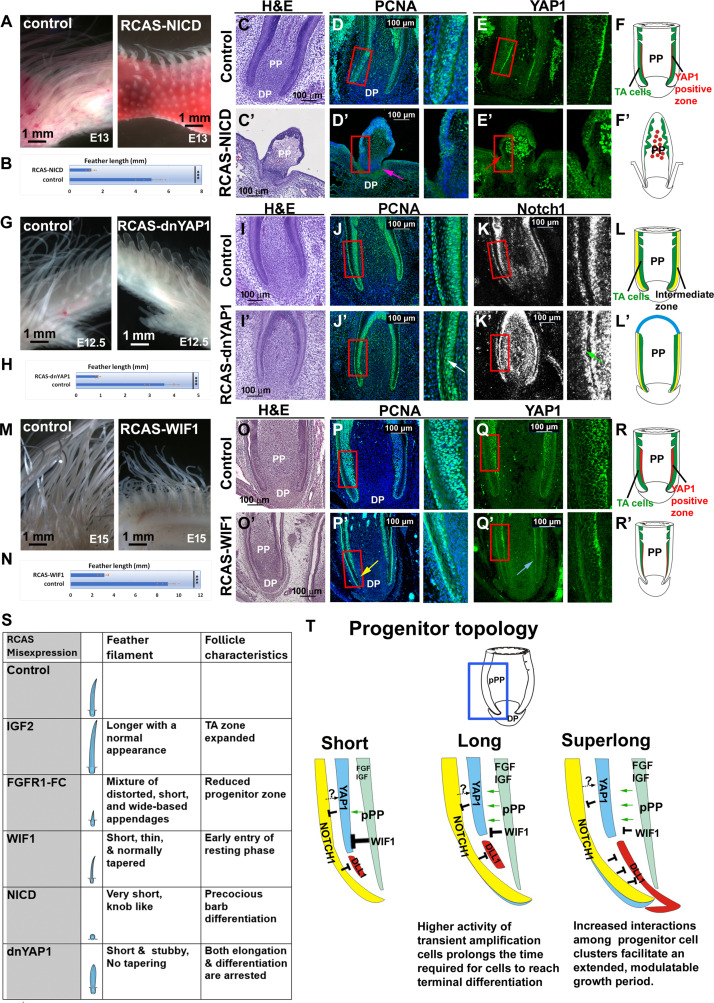
Functional study and summary of progenitor topology in the regulation of feather lengths. (**A** and **B**) Disruption of Notch signaling by misexpression NICD shows short feather filaments. (**C** to **F′**) Comparison of H&E, PCNA, and YAP1 staining between control and RCAS-NICD–infected samples. Note the precocious barb ridge formation (C′) and the lack of PCNA-positive cells in the epidermis (D′) (pink arrow). RCAS-NICD samples show the disappearance of YAP1 in the epidermis (E′) (red arrow) but a paradoxical increase in YAP1 in the PP. (**G** and **H**) Inhibition of YAP activity converts the tapering feather filament into short, stubby, cylindrical filaments. (**I** to **L′**) Comparison of control and RCAS-dnYAP1–infected samples. Note the reduced thickness of TA cell zone (J′) (white arrow) and intermediate layer (K′) (green arrow). There are large numbers of NOTCH1-positive PP cells. The distal end fails to form barb branches. (**M** and **N**) Inhibition of WNT signaling by RCAS-WIF1 reduces feather length. (**O** to **R′**) Comparison of control and RCAS-WIF1–infected samples. Note the reduced feather follicle width (O′), the reduction of PCNA-positive cells in the epidermis (P′) (yellow arrow), and reduced YAP1 expression (Q′) (blue arrow). (**S**) Table summarizing the findings using RCAS-mediated gene misexpression. (**T**) Model of YAP1 interacted with the DLL/NOTCH pathway regulating feather length by fine-tuning the progenitor cell topology. Compared to short feathers, long feathers have a bigger stem cell zone marked by DLL1 expression (red color). The Phoenix chicken main sickle feathers have an elongated stem cell zone and higher YAP1 activity that may contribute to the longer growth period. Left: Schematic drawing of a feather follicle. For [(B), (H), and (N)], five flight feather filaments from the right (experiment) and left (control) wings were measured. ***P < 0.001.

To investigate their functions, we examined NOTCH pathway function using a constitutively active form of NOTCH1 (Notch intracellular domain; RCAS-NICD) (*40*) to interrupt the NOTCH activity. We injected these plasmids to the right-wing bud at E4. The flight feather follicle in the right wing shows decreased feather length (Fig. 7, A and B; *n* = 5/5 embryos). The perturbed follicle shows decreased proliferating cells in the proximal feather follicle (Fig. 7C′ compared to Fig. 7C; Fig. 7D′, pink arrow). Barb ridges form precociously, and feather elongation is halted, probably because of depletion of progenitor cells. *YAP1* expression in the epidermal progenitor zone is reduced but is increased in the PP (Fig. 7E′, red arrow). The results suggest that homeostatic equilibrium within the feather progenitor zone is disrupted, with precocious barb differentiation and depletion of progenitor cells (Fig. 7, F′ versus F; Fig. 7S).

To test the functionality of YAP1 in feather follicle development, we constructed a dominant-negative form of YAP1 (RCAS-dnYAP1). The injected wing shows a shortened feather filament, but they are stubby and cylindrical and lack the normal tapering distal end (Fig. 7, G and H; *n* = 6/6 embryos). Tissue section analyses showed that in the follicle base, the TA cell zone thickness is reduced (Fig. 7J′, white arrow, compared to the control in Fig. 7J). *NOTCH1* expression is reduced in the intermediate layer but greatly increased in dermal PP cells (Fig. 7K′, green arrow). The most distinctive phenotype is the absence of barb branch formation at the distal end, resulting in a flat-topped structure without tapering (Fig. 7, L′ versus L; Fig. 7S).

The function of WIF1 (a WNT pathway antagonist) was tested by injecting RCAS-WIF1 into the developing flight feather follicles. The perturbed feather filaments are shorter and thinner but still have a tapered distal end (Fig. 7, M and N; *n* = 4/5 embryos). Sections show that the follicle size is reduced (Fig. 7O′). The epidermis adjacent to the DP exhibits a loss of PCNA-positive cells (Fig. 7P′ compared to Fig. 7P, yellow arrow). *YAP1* expression in the progenitor zone is also reduced in the proximal follicle (Fig. 7Q′ compared to Fig. 7Q). However, the proportion of different progenitor cells remains nearly normal, suggesting that WIF1-treated feather follicles enter the resting phase prematurely (Fig. 7, R′ versus 7R; Fig. 7S).

We then used RCAS-Wnt3a to test the possible feedback regulation of the Wnt/WIF1 axis. The RCAS-Wnt3a–treated sample shows short and fat feather filaments (fig. S13, A to F′; *n* = 4/4). The PCNA-positive zone is only restricted in the base the epidermis in the treated sample (fig. S13C′, green arrow). The *YAP1* expression zone is also reduced (fig. S13D′, red arrow). In the normal embryonic flight feather, *WIF1* is expressed in the pPP (fig. S13E). The RCAS-Wnt3a–treated sample shows the faint *WIF1* in the tip of feather PP (fig. S13E′, blue arrow). This result suggests that balance of Wnt and WIF1 signaling is crucial for feather morphogenesis. The feedback regulation of Wnt/WIF1/YAP1 would be worth investigating further in the future.

These functional studies showed the importance of YAP / NOTCH / WNT signaling for maintaining the homeostasis of progenitor cells/TA cells/intermediate layer cells, which then provide a continuous supply of epidermal cells for feather elongation. YAP activity is also important to regulate NOTCH signaling. Together, they play critical roles for maintaining progenitor cell zone homeostasis and growth period duration.

## DISCUSSION

The summary of this study is as follows: (i) We examine the topological distribution and size of cell clusters, including progenitor, proliferating, apoptotic, and differentiated cell zones in contour, lesser sickle, and main sickle feather follicles. (ii) Through transcriptomic profiling across early growth, late growth, and resting stages, we identify molecular signals with trends of up- or down-regulation, pinpointing candidate pathways that enhance or limit feather growth. (iii) Using scRNA-seq, we map cellular clusters within the feather collar regions. CellChat analysis reveals that FGF and IGF signaling activities are elevated in longer feathers, while pseudotime analysis suggests that progenitors in short feathers differentiate faster than those in long feathers. Gene misexpression experiments using RCAS demonstrate that IGF and FGF pathways act as activators for feather growth. (iv) In Phoenix chickens, characterized by superlong main sickle feathers, we observe intensified cell interactions and expanded progenitor cell and intermediate cell zones. (v) Misexpression of NICD (active NOTCH1 intracellular domain) induces premature barb ridge differentiation, while dominant-negative YAP1 causes the collar to remain in the intermediate layer stage. These results suggest that YAP1 and NOTCH1 play crucial roles in maintaining homeostatic growth and differentiation, regulating the duration of feather growth.

### scRNA-seq analyses identify distinct cell clusters in feathers follicles

We identified several major zones in the collar with different cellular activities, including the stem cell zone defined by label retention, the progenitor cell zone which differentiates to form feather branches, and the apoptosis zone (Fig. 1N). We used double TA cell labeling to identify progenitor cells and the route of their expansion and differentiation. Our result showed the dual fates of epidermal progenitors: One group differentiated to form the apoptosis zone and became the feather sheath; the other group formed the basal layer, which proliferated and migrated from the collar to eventually form the feather branch (Fig. 1T).

We found different configurations of the LRC, TA, and differentiation cell zones in the feather follicles with different lengths (Fig. 2O). We showed that both IGF and FGF pathways play critical roles in feather length control. We propose that the IGF pathway regulates feather size by stimulating cell proliferation in the progenitor cell activity, whereas FGF is needed for maintaining the feather growth during the growth phase. When FGF signaling is interrupted, the feather enters the resting phase precociously (Fig. 5I).

Our evidence suggests that the control lies in interactions between the epidermal stem cells and their dermal niche. We have revealed some interesting molecular interactions between the epidermal stem cells and their niche, but how the niche provides this information remains unknown and warrants further study.

### Wnt/IGF/FGF/BMP signaling regulates feather length by modulating progenitor proliferation/differentiation

#### IGF and Wnt as activators

Wnt signaling has been shown to be involved in initiating the growth phase of feather cycling (*41*). Here, we show that IGF signaling promotes feather growth. Elevated IGF1 has been shown to be associated with initiating the growth of more feathers in wild juvenile bearded reedlings (*42*), implying the involvement of IGF signaling in overall feather growth.

#### *FGF as modulators*, *depending on members*

Previously, we found that FGF signaling is involved in adult feather follicle collar epidermis development (*24*). Overexpression of *Spry4*, an FGF inhibitor, promotes the formation of distal feather follicle components, while diminishing the collar epithelium. In contrast, overexpression of *Fgf10* promotes expansion of proximal follicle structures and inhibits distal branching (*24*). FGF pathway members show bipolar distribution in the feather epidermal progenitor cells: Some are high in early growth phase (*FGF6*, *FGF9*, *FGF10*, *FGFBP1*, *FGFR1*, *MAPK1*, and *SRC*), while some are high in the resting phase (e.g., *FGF1*, *FGF2*, *FGF18*, *FGF23*, *FGFR2*, and *FGFR3*) (fig. S3B). This implies the complex interactions among FGF pathway signaling, and future studies will be required to sort out their interactions.

#### BMP and WIF1 as inhibitors

Bulk RNA-seq reveals that this progenitor cell zone is enriched in BMP and WNT antagonist when entering the resting phase of the feather cycle (Fig. 3F). In human hair, *WIF1* is expressed in the interfollicular epidermis and plays a critical role in suppressing keratinocyte proliferation (*43*). Our functional study shows that the progenitor cell zone adjacent to the DP in *WIF1*-overexpressed samples loses proliferation activity and mimics progenitor cells in the resting phase (Fig. 7P′).

scRNA-seq enables the analysis of intrasample heterogeneity and cell-cell interactions (*44*). Using this technology, we found cell heterogeneity, cell-cell interactions, and active pathways in feather follicles. We identify different progenitor types within distinct epidermal cell clusters. We found different cell cluster configurations between short and long feathers. Pseudotime analysis of epidermal cell state transitions reveals that progenitor cells in long feathers undergo a longer route to attain terminal differentiation (Fig. 5B), which is further supported by a stronger IGF and FGF signaling (Fig. 5N).

### Enhanced Notch/DLL1/YAP signaling modulates superlong feather progenitor population size

There are several chicken strains that have long tails, including Onagodori, Shokoku, Phoenix, etc. We used Phoenix chickens as a model to study growth control in long tail feathers. Main sickle feathers in male Phoenix chickens can grow up to 1 m long. These feathers are approximately three times longer than White Leghorn chicken main sickle feathers. Our study revealed an altered spatial organization of the progenitor cell niche in the Phoenix main sickle feather follicles (Fig. 6F). We also found that *YAP1* and *DLL1* are adjacently expressed in different domains of the feather progenitor cell zone. In Phoenix main sickle feathers, the *DLL1* domain is expanded, whereas the *YAP1* expression domain is also enhanced (Fig. 6J and table S6). These distinctive characteristics may increase the interactive surface areas, allowing for enhanced modulation possibilities and recruitment of progenitor cells, thereby supporting prolonged feather growth.

To investigate their functions, we used RCAS to misexpress NICD to activate Notch signaling and RCAS-dnYAP1 to inhibit YAP1 signaling. When NOTCH pathway activity is enhanced using RCAS-NICD, feathers undergo precocious barb ridge differentiation and depletion of progenitor cells (Fig. 7, F′ and S). When RCAS-dnYAP1 was introduced, both feather elongation and barb differentiation were arrested, resulting in the formation of a uniquely cylindrically shaped appendage (Fig. 7, L′ and S). These results suggest an interplay between the YAP and NOTCH pathways in maintaining the intermediate layer and growth period. Thus, YAP1 and NOTCH1 may form a feedback loop, ensuring the persistence of both pathways during the growth phase. The gradual decline of YAP activity and the rise of WIF1 in adjacent dermis eventually ends the growth phase and moves the follicle toward the resting phase.

Perturbation experiments disrupting these interactions led to shorter feather phenotypes. Analyses of the follicles revealed distinct modes of arrest: the presence of abnormally large progenitor cells unable to differentiate properly, premature differentiation of feather keratinocytes, or early entry into resting phases. These observations provide valuable insights into the sequential processes governing feather length control, offering a foundation for dissecting the intricate mechanisms underlying this regulation.

### Comparing hair and feather length control

As stated in the Introduction, mammalian hair length is regulated by interactions among Wnt, IGF, BMP, and FGF pathways. In hair, *FGF7* and *FGF10* have been shown to promote anagen onset and maintenance, resulting in longer hairs (*15*), while *FGF18* enforces telogen and shortens overall hair growth (*45*). Here, epidermal progenitor cell bulk RNA-seq across feather growth stages shows that *FGF10* is also increased in the growth phase and *FGF18* is high in the resting phase (fig. S3B). We also showed that *FGF10* can increase the size of the progenitor zone (*24*). *FGF5* is not highlighted in this analysis. We did not find its expression in feathers. While we did not evaluate *FGF18* directly, it is possible that they are also involved in maintaining the resting phase in feather growth. It is intriguing that *FGF1* and *FGF2* are also high in the resting phase of feather cycling. Because feathers and hair arose through convergent evolution (*18*), they need not rely on identical signaling pathways to control growth.

YAP1 plays an important role in skin and hair follicle development, stem cell activation, regenerative processes, and aging. The nuclear localization of YAP diminishes with age and is linked to the reduced proliferative capacity of epidermal progenitor cells. In addition, YAP stimulates the growth of basal epidermal progenitor populations, drives cell proliferation, and prevents their full differentiation (*46*). Cross-talk between the Hippo/YAP and WNT/β-catenin signaling pathways has been implicated in maintaining epidermal homeostasis. Studies have demonstrated that heightened YAP activity in basal keratinocytes of murine skin triggers β-catenin activation (*47*), leading to pronounced epidermal hyperplasia in both the interfollicular epidermis and hair follicles (*48*, *49*). Our study on feather length regulation also highlights the interaction among NOTCH, WNT, and YAP pathways, which may be crucial for precise feather morphogenesis and size control.

### Summary, limitation, and future directions

In summary, here, we did a molecular characterization of the classical “feather collar” (*22*). While it remains true that the collar drives feather growth (*23*), we now show that they are composed of progenitor cells, and we also use scRNA to identify the stem cells, TA cells, intermediate layer, and differentiated cells including the barb ridges and feather sheath. In feathers of varying lengths, we observed that the relative size of these cell clusters and the duration of their active phases during feather cycling can differ. Our experimental data suggest two mechanisms that regulate feather length. One is IGF/FGF signaling. IGF signaling activates TA cell proliferation and governs the growth rates of the feather filament. The FGF pathway is involved in driving and ending the growth phase in a pathway member–dependent way, and further studies will be required to sort out the specific function of different FGF members. The other is YAP/NOTCH/DLL1 interactions, affecting the size and half-life of progenitor cell clusters and thereby regulating the feather growth period. These results set up the cellular and molecular players regulating feather length. These factors collectively fine-tune the topology of progenitor cells within the feather follicle, ultimately determining the length of the feather (Fig. 7T). With the reorganization of progenitor cell clusters, the superlong Phoenix sickle feathers exhibit a growth period up to three times longer than White Leghorn sickle feathers, extending the growth period by nearly 8 months.

Our findings demonstrate that feather length is controlled by a combination of growth rate and growth duration. Growth rate can be modulated by adjusting tyrosine kinase receptor activity, while extending the growth period depends on regulating the balance and communication among progenitor cell clusters. With this repertoire of potential controlling steps, different feathers, whether from different body regions or different variants, use distinct strategies to regulate these processes. Together, this study not only provides insights into feather length regulation but also offers a framework for understanding how organ size can be finely tuned by coordinating stem cell dynamics and growth signaling pathways.

## MATERIALS AND METHODS

### Ethics statement

All the animals used in this study were processed following an approved protocol (11903) of the Institutional Animal Care and Use Committees of the University of Southern California (Los Angeles, CA).

### Chickens

White Leghorn chickens were hatched from pathogen free fertilized eggs (SPAFAS, Preston, CT). Chickens were raised at 22°C with 12-hour cycles of light and darkness. Six-month to 1-year-old male chickens were used in this study. Phoenix chicken eggs were purchased from Chickweed Farms LLC.

### Feather length and growth rate measurement

Resting phase contour, main sickle, and lesser sickle feathers were plucked, and their full lengths were measured. Feather width was measured at the widest feather section. To calculate the growth rate, the length of regenerated feathers was measured every week. All statistics were based on the measurement of at least three feathers. Lesser sickle feather no. 3 was used to represent lesser sickle feathers.

### BrdU, IdU, and CldU pulse labeling

Four 1-year-old male White Leghorn chickens were used in this study. For TA cell pulse labeling, adult chickens were injected with BrdU (Sigma-Aldrich; 2 mg/kg), and feathers were collected after 2 hours. For TA cell double labeling, IdU (Sigma-Aldrich; 2 mg/kg) was injected. After 22 hours, CldU (Sigma-Aldrich; 2 mg/kg) was injected, and feather follicles were collected at 24 hours.

### LRC and TA cell double labeling

Three 1-year-old male White Leghorn chickens and three 1-year-old male Phoenix chickens were used in this study. Resting phase contour, lesser sickle, and main sickle feathers were plucked. After allowing the feathers to regenerate for 1-week, 50 ml of IdU (0.1% in water) was added to the drinking water daily for 1 week. After a 2-week chase period, CldU (2 mg/kg) was injected, and feather follicles were collected 2 hours later. This strategy allows us to examine both LRCs and TA cells labeled with IdU and CldU, respectively, in the same feather follicle. For each feather type, at least three feather follicles were collected from different chickens.

### Paraffin section and immunostaining

Feather follicles were fixed in 4% paraformaldehyde at 4°C overnight, and 7-μm longitudinal paraffin sections were prepared according to standard procedures. H&E and immunostaining were followed by procedures previously described in (*25*). We used the following antibodies: β-catenin (Sigma-Aldrich, C7207; 1:200), ITGA6 (Developmental Studies Hybridoma Bank, P2C62C4; 1:10), p63 (Santa Cruz Biotechnology, sc-25268; 1:200), PCNA (Millipore, MAB4078; 1:200), and cytokeratin 17 (KRT17. K17, Abcam, ab53707; 1:200). β-Keratin antibody is from R.H. Sawyer.

For BrdU staining, sections were treated with 0.01 M citrate buffer (pH 6.0) by microwaving for 6 min. BrdU was detected by mouse anti-BrdU (BD Biosciences, 347580; 1:200). Anti-mouse Alexa Fluor 488 (Invitrogen, A11029; 1:200) was used as a secondary antibody. DAPI was used to visualize nuclei.

For IdU/CldU staining, sections were treated with 0.01 M citrate buffer (pH 6.0) by microwaving for 6 min. CldU was detected using a rat anti-BrdU antibody (BU 1/75, Abcam, Ab6326-250; 1:200); anti-rat Alexa Fluor 488 (A11006; 1:200) from Invitrogen was used as the secondary antibody. IdU was detected by mouse anti-BrdU (BD Biosciences, 347580; 1:200); anti-mouse Alexa Fluor 546 (A11030; 1:200) from Invitrogen was used as the secondary antibody. Fluorescent imaging was performed using Keyence BZ-X710 microscope and Leica TCS SP8 confocal microscope.

### TUNEL assay

TUNEL assay was performed by using In Situ Cell Death Detection Kit, Fluorescein (Roche, 11684795910) according to the manufacturer’s protocol.

### Section in situ hybridization

To generate RNA probes, polymerase chain reaction (PCR) was performed using cDNA from embryonic day 8 chicken skin. PCR primers were as follows: *IGFBP5*, ctcagcgagaagagctaccg (forward) and gacttcactccacgttgctg (reverse); *FGFBP1*, ggctgaatcaaacccagaga (forward) and gcactttttgtcttgcacca (reverse); cysteine and glycine rich protein 2 (CSRP2), aacaaatgtggtgcctgcg (forward) and ctcctgctccctgaccatag (reverse); epidermal differentiation protein (EDPE), cctgaacctgcaaagtgtcc (forward); gcttttctcttcggggcttc (reverse). PCR products were inserted to p-drive (QIAGEN) to make antisense RNA probes. *Feather keratin* and *scale keratin* probes were from (*50*). Section in situ hybridization was performed according to procedures described in (*50*). Diluted eosin was used for faint counterstaining.

### RNAscope analysis

RNAscope was performed using the Multiplex Fluorescent v2 system [Advanced Cell Diagnostics (ACD), 323100]. The standard RNAscope protocol was used according to the manufacturer’s instructions. We used the following probes: *LGR5* (catalog no. 480781-C1), *YAP1* (catalog no. 1325011-C1), *DLL1* (catalog no. 1325021-C2), *NOTCH1* (catalog no. 458901-C3), *TNC* (catalog no. 1055441-C2), and *NCAM* (catalog no. 1055371-C3).

### Bulk RNA-seq for epidermal progenitor cell zone and whole feather follicles

For epidermal progenitor cell zone, early growth phase (regenerated 3 weeks), late growth phase (regenerated 7 weeks), and resting phase contour feathers (regenerated 10 weeks) were used. Feathers were plucked and the DP was dissected. Papilla ectoderm was removed from the DP after treatment with calcium- and magnesium-free saline (2× CMF) with 0.1% EDTA on ice for 20 min. Two replicates from each stage were used.

Total RNA was isolated using TRIzol (Thermo Fisher Scientific, 15596026). The RNA quantities and qualities of each individual were analyzed using a NanoDrop (Thermo Fisher Scientific) and BioAnalyzer II (Agilent Technologies). We used 1 μg of total RNA from each sample to construct an RNA-seq library using TruSeq RNA sample preparation v2 kit (Illumina). Sequencing (50 cycles of single read) was performed using HiSeq 2000 at the USC Epigenome Center. For the whole feather follicles, a polyadenylate enrichment + optimized NEB Ultra II directional kit was used to generate RNA-seq library and sequenced using NovaSeq 6000 PE150 by Novogene.

### Bulk RNA-seq: Read count normalization and DEG identification

The read counts and transcript per million (TPM) values for each gene were obtained from the mapping files using StringTie (*51*, *52*) using default parameters and the genome annotation file (GRCg6a, Ensembl). Genes with mean TPM value across libraries higher than 1 were defined as the expressed genes. Read counts among the libraries were normalized, and DEGs were calculated using DESeq2 (*53*). The expressed genes were defined as DEGs when their log_2_ fold change of >2 and *q* < 0.01 between the comparisons (table S1). DEGs and their normalized log-transformed values were calculated by DESeq2 and used as the input for the following analysis. The hierarchical clusters and heatmaps were generated from the merged DEGs by the R package “pheatmap.”

For the IGF and FGF pathway heatmap in fig. S3, genes from the IGF and FGF pathways were obtained from the human gene sets in the Molecular Signatures Database (*54*). To perform gene ontology analysis on the DEGs, the g:GOSt tool embedded in g:Profiler, a public web server for characterizing and manipulating gene lists derived from high-throughput genomic data, was applied to each DEG set (*55*). The mouse database was used to increase the sensitivity of the analysis, and the Benjamini-Hochberg false discovery rate threshold was set to less than 0.05 (table S2).

### Single-cell RNA sequencing

Two contour feathers and one lesser sickle feather were used from White Leghorn chickens. Two main sickle feathers were used from Phoenix chickens. Regenerating feather follicles was surgically dissected, and only 0.5-cm feather follicles with DP and DS were used for scRNA-seq (Fig. 4A). Feather follicles were cut to small pieces and incubated in 5 ml of RPMI 1640 solution containing 232 U of deoxyribonuclease I (Sigma-Aldrich), 1.25 mg of Liberase (Sigma-Aldrich), and 5 mg of dispase:collagenase (Sigma-Aldrich) for 2 hours at 37°C and quenched with fetal bovine serum. Cells were passed through a 70-μm filter and centrifuged at 500*g* for 5 min, and the pellet was resuspended in phosphate-buffered saline–free of Ca^2+^ and Mg^2+^ and 1% bovine serum albumin (BSA). Dead cells were removed using the Dead Cell Removal Kit (Miltenyi Biotec). Live cells were resuspended in 0.04% UltraPure BSA (Sigma-Aldrich) and counted using the automated cell counter Countess (Thermo Fisher Scientific). Cells were captured using Chromium (10x Genomics). GEM (Gel Bead-In EMulsions) generation, barcoding, post–GEM–RT (reverse transcription) cleanup, cDNA amplification, and cDNA library construction were performed using Single-Cell 3′ v2 chemistry (10x Genomics). cDNA libraries were sequenced on an Illumina HiSeq4000 platform (Illumina) (one lane, 100 paired end). Cell counting, suspension, GEM generation, barcoding, post–GEM-RT cleanup, cDNA amplification, library preparation, quality control, and sequencing were performed at the Genomics High-Throughput Sequencing facility at the University of California, Irvine.

### scRNA-seq raw data processing

Preprocessed digital matrices from individual feather follicle datasets were processed using Seurat (version 4.0.1) (*56*). Seurat objects were created and log normalized with a scale factor of 10,000. Variable features were identified using vst with top 2000 features. Data were scaled, and metadata variables, including mitochondrial gene expression, were regressed to select cells with 100 to 4000 RNA features and less than 10% of mitochondrial counts. Principal components analysis was calculated using variable features identified using a combination of heuristic and statistical approaches. The first 20 principal components analysis dimensions were used to compute cell neighbors and Uniform Manifold Approximation and Projection (UMAP). Individual datasets were visualized using a two-dimensional embedding. Individual datasets from White Leghorn chicken contour (short feather) and lesser sickle feather (long feather) were processed for integration, downstream analyses, or visualization with Seurat’s FindIntegrationAnchors and IntegrateData functions. Two Phoenix chicken main sickle feather (superlong feather) datasets were combined to match the cell number of White Leghorn chicken feathers.

### CellChat analysis

CellChat (*35*) was used to compare the strength of the interactions between short and long feathers. All parameters for the analysis were set to their default value.

### Gene signatures for IGF and FGF pathways

The strength of IGF and FGF intracellular signature was obtained by computing the average, normalized gene expression of the gene sets in individual cells. Then, the IGF/FGF strength was averaged over clusters for the epidermal cell states. The correlation between IGF and FGF signatures was computed on all epidermal cells. The nonoverlapping correlation was computed by first removing genes that are shared between the IGF and FGF gene sets and then repeating the steps previously described.

### Inference of GRN

Inference of the GRN was performed with spliceJAC (*36*). Briefly, spliceJAC uses the unspliced and spliced RNA counts from the scRNA-seq dataset to predict a directed gene GRN. The set of node genes in the GRN was built by taking the top 200 highly variable genes identified in the epidermal cells as well as the IGF and FGF signature genes to specifically predict IGF/FGF-mediated gene regulation. The package was otherwise run with default parameter settings. The 200 top variable genes were selected using ScanPy’s highly variable gene selection feature (*33*). The gene centrality score in the predicted GRN was calculated using spliceJAC’s built-in betweenness centrality function.

### Pseudotime and trajectory analysis

Pseudotime analysis was performed using ScanPy’s built in diffusion pseudotime function (*33*) on the preprocessed and well-annotated dataset with default parameters. A randomly chosen cell in the epi–progenitor 1 cluster was selected as root for pseudotime calculation. The transition probability between epidermal cell states in the short and long feather datasets were computed using PAGA (*34*) with default parameters.

### RCAS infection in chicken embryo and adult feather follicle

RCAS-insulin like growth factor 2 (IGF2) plasmid was obtained from Fults and co-workers (*57*). The secreted dominant-negative version of FGFR1 (RCAS-FGFR1-Fc) was from Mandler and Neubüser (*37*). The RCAS-NICD was from (*40*). RCAS-FGF8 and RCAS-Wnt3a were provided by Tabin laboratory. RCAS-Sprouty4 was from Martin laboratory (*38*).

RCAS-WIF1 was constructed by ligate the PCR product (forward, 5′-AAATATGCGGCCGCACCTGAATGGCCGCGGCGGG-3′; backward, 5′-GCACTAGTCCAGATATAATTGGATTCGGG-3′) to an RCAS vector carrying an mCherry fluorescent tracer (RCAS-2A-mCherry). 

For the RCAS–dominant negative form YAP1 (RCAS-dnYAP1) cloning, plasmid pLX304-YAP1 _60-89, encoding dominant-negative YAP1, was obtained from Addgene (W. Hahn’s group). The YAP1 _60-89 sequence was amplified using the forward primer 5′-ACTCTGCTGGCGGCCAGCAGAGCTCTCTGGCTACTGT-3′ and reverse primer 5′-CTCTGCCCTCACTAGCGTAGAATCGAGACCGAGGAGA-3′ and inserted into the RCAS-2A-mCherry plasmid using In-Fusion method.

To make RCAS–dominant negative form IGF1R (RCAS-dnIGF1R), we used the following primers (forward, 5′-ACTCTGCTGGCGGCCGCGCCACCATGAAGTCTGGCGCTGGAGG-3′; backward, 5′-CTCTGCCCTCACTAGTAGAGGCTCTCTCCCCATTGT-3′). This PCR product includes IGF-1R residues 1 to 486, encoding soluble receptor (*58*).

RCAS viruses were prepared and concentrated at 20,000 rpm for 2 hours at 4°C. We injected the RCAS virus (1 × 10^7^ IU/ml) to the right forelimb bud at E4 and collected embryos at E12 to E15. RCAS-GFP virus was used as control, and six embryos at E13 were collected.

### Statistical analysis

In Figs. 2 (A, B, D, G, I, J, L, and N), 3 (D to F), 5 (E and J), 6 (B and E), and 7 (B, H, and N), data are means ± SD. All comparisons were made by applying a paired two-tailed Student’s *t* test.
