# Supplementary material for: Regulation of feather length: FGF/IGF signaling and NOTCH/YAP modulation of progenitor cell topology
Source: Sci Adv. 2025 Aug 22;11(34):eadw2382. doi: 10.1126/sciadv.adw2382 (PMC12372877; doi:10.1126/sciadv.adw2382)
Supplement: Supplementary file 1 — Figs. S1 to S13 Legends for tables S1 to S5 Table S6 [file sciadv.adw2382_sm.pdf]

Science Advances
AAAS

Supplementary Materials for

Regulation of feather length: FGF/IGF signaling and NOTCH/YAP modulation of progenitor cell topology

Ping Wu *et al.*

Corresponding author: Cheng-Ming Chuong, cmchuong@usc.edu; Ping Wu, pingwu@usc.edu

Sci. Adv. **11**, eadw2382 (2025)
DOI: 10.1126/sciadv.adw2382

The PDF file includes:

Figs. S1 to S13
Legends for tables S1 to S5
Table S6

Other Supplementary Material for this manuscript includes the following:

Tables S1 to S5

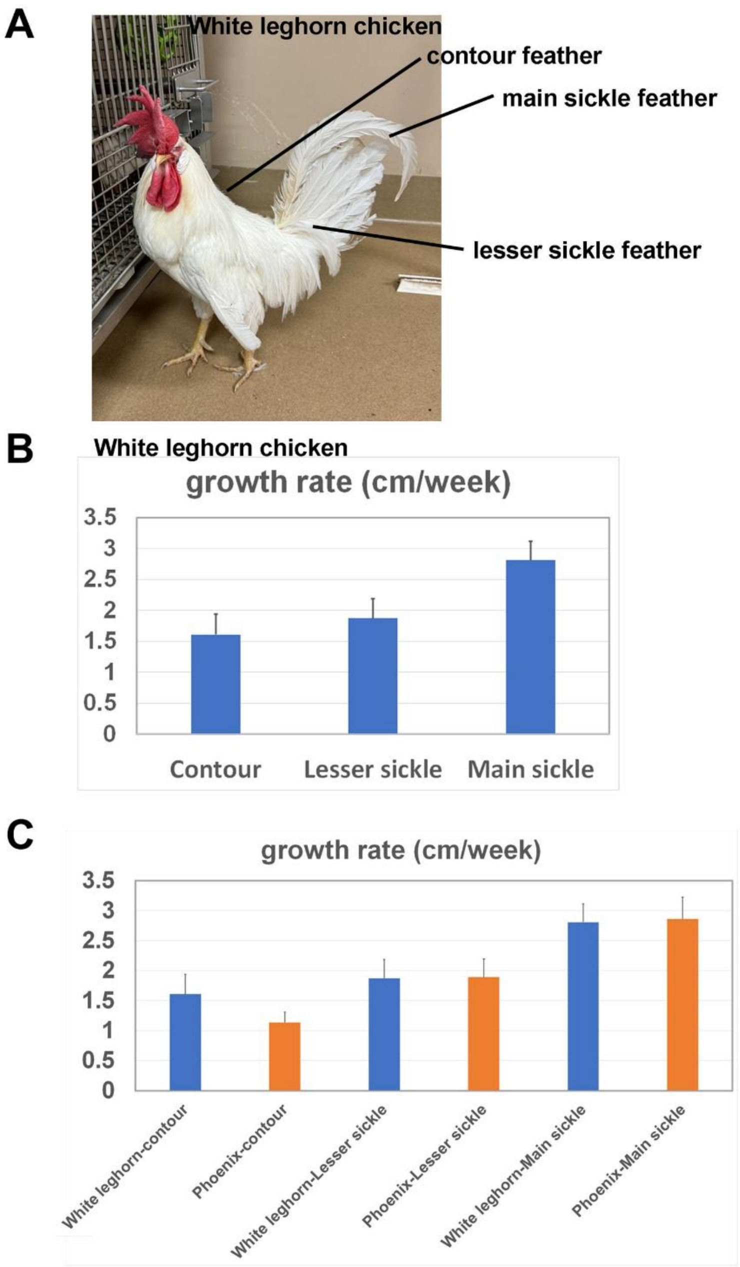

Figure S1. Growth rate of feathers

A. White leghorn chicken showing contour, main sickle and lesser sickle feathers.

B. Growth rate comparison of White leghorn chicken contour, lesser sickle and main sickle feathers.

C. Growth rate comparison of White leghorn and Phoenix chicken contour, lesser sickle and main sickle feathers.

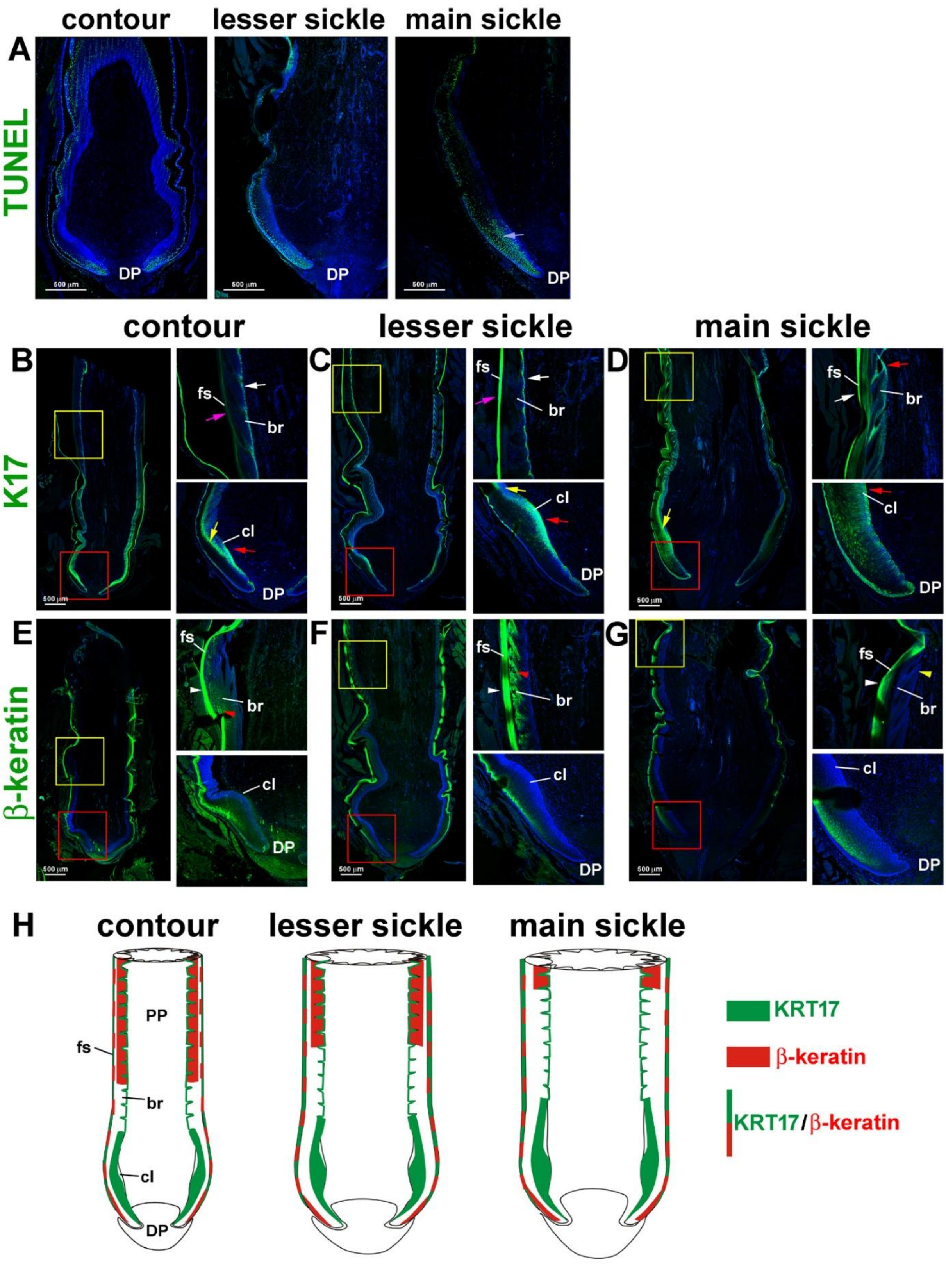

contour
lesser sickle
main sickle
A
TUNEL
DP
DP
DP
500 µm
contour
lesser sickle
main sickle
B
C
D
K17
fs
br
cl
DP
E
F
G
β-keratin
H
contour
lesser sickle
main sickle
PP
fs
br
cl
DP
KRT17
β-keratin
KRT17/β-keratin

Figure S2. The apoptosis zone and expression of K17 and β-keratin in feather follicles.

A. TUNEL staining of early growth phase contour, lesser sickle and main sickle feather follicles from White leghorn chicken. Blue arrow indicates the expanded apoptosis zone in the main sickle feather follicle. B-D. K17 expression. E-G. β-keratin expression. The red and yellow box in each panel shows the follicle base and the differentiating barb ridges, respectively. In B-D, the red arrow indicates the K17 expression in the collar epidermis. The yellow arrow indicates the K17 expression in the intermediate layer. The white arrow indicates the K17 expression in the basal layer cells of the barb ridge. The pink arrow indicates K17 expression in the feather sheath. In E-G, the white arrowhead indicates the β-keratin expression in the feather sheath. The red arrowhead indicates the β-keratin in the barb ridge of contour and lesser sickle feathers. In contrast, the main sickle feather did not express β-keratin at a similar depth of the feather follicle (yellow arrowhead). H. Schematic drawing summarizing the K17 and β-keratin immunostaining.

br, barb ridge; cl, collar; DP, dermal papilla; fs, feather sheath. PP, pulp.

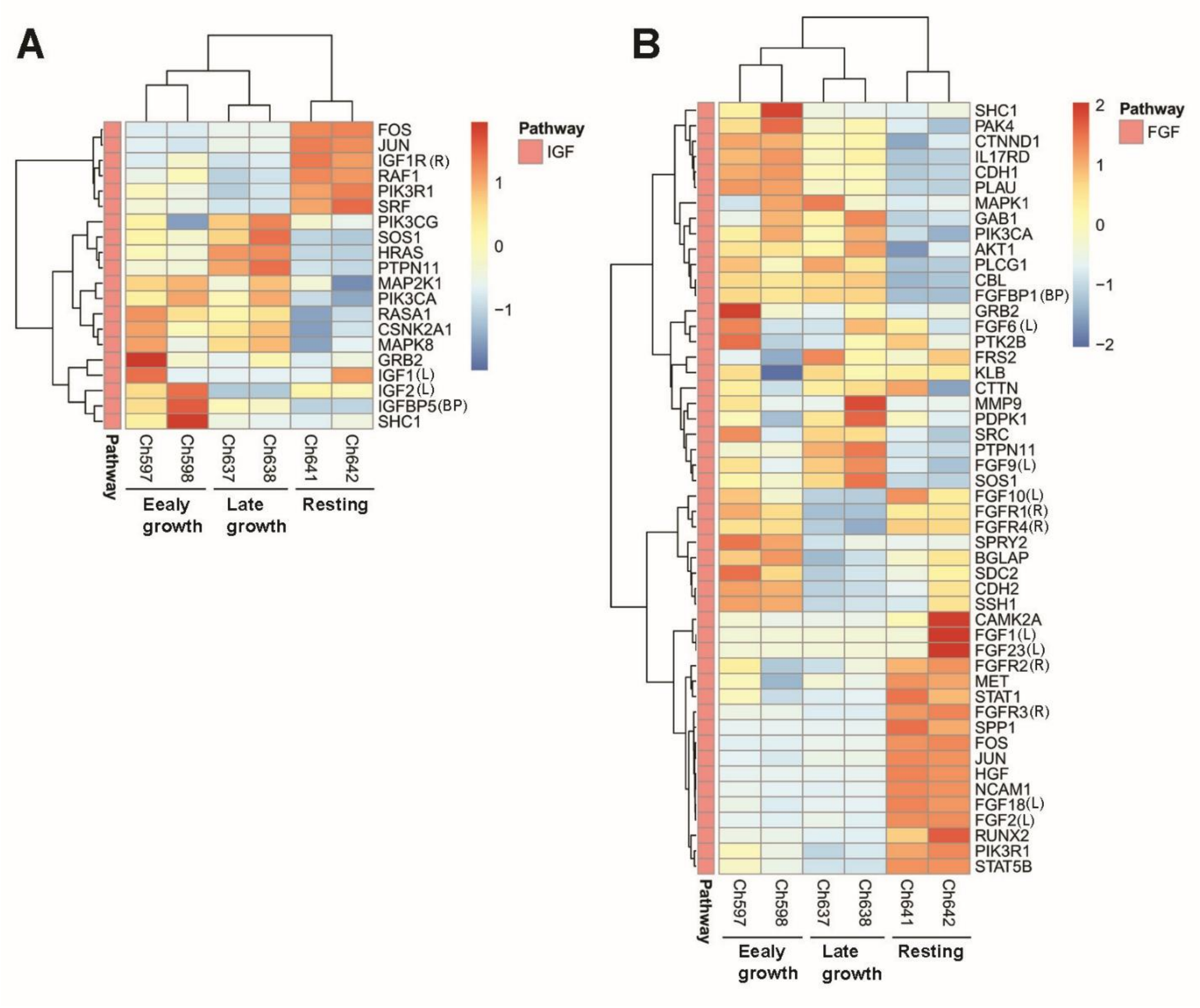

Figure S3. The gene expression analysis of IGF and FGF pathways in feather progenitor cell zone.

A. FGF pathway. B. FGF pathway. Genes from the IGF and FGF pathways were obtained from the human gene sets in the Molecular Signatures Database (MSigDB). Gene TPM values were row-scaled and represented across three stages: early growth phase, late growth phase, and resting phase.

BP, binding protein; L, ligand; R, receptor

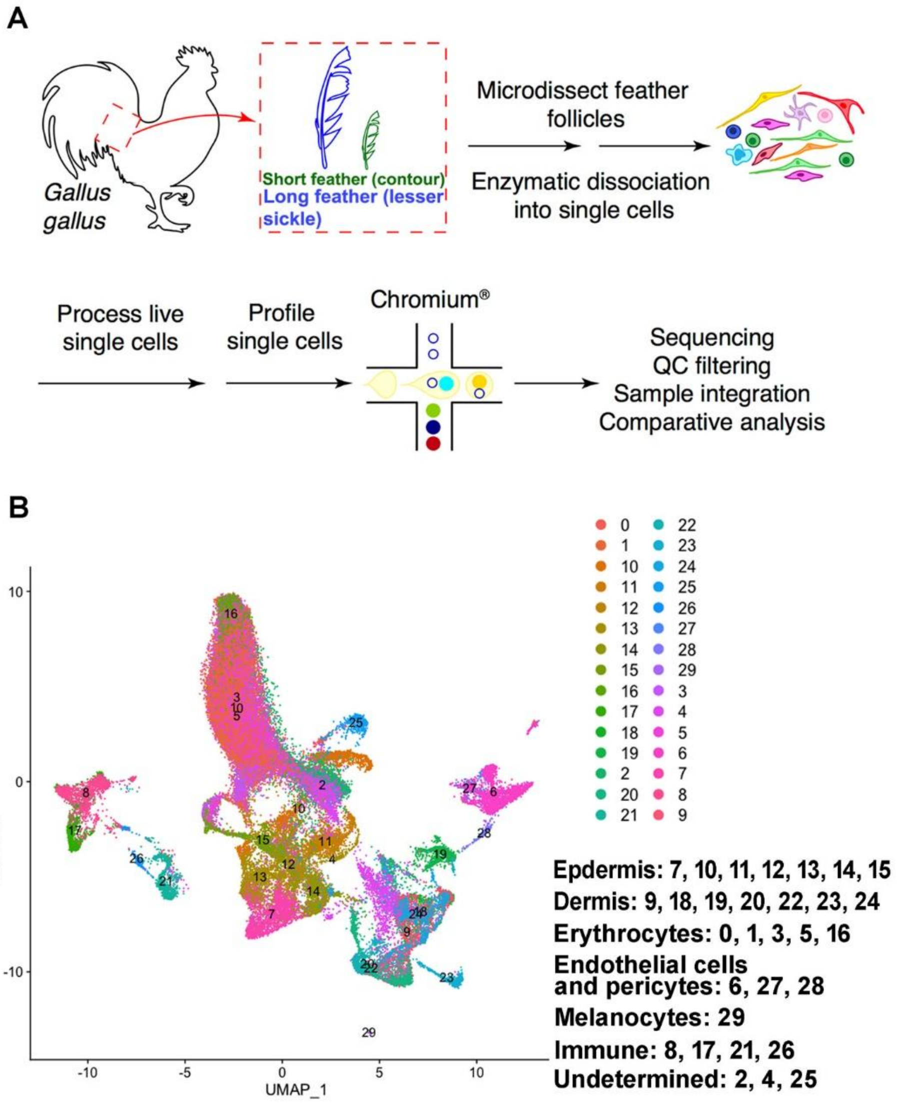

Figure S4. Feather follicles single cell RNA-seq analysis

A. Sample preparation for scRNA-seq of regenerated feather follicles from White leghorn chicken.

B. 30 cell clusters are identified. The cells from contour (short) and lesser sickle feather (long) are merged in this panel.

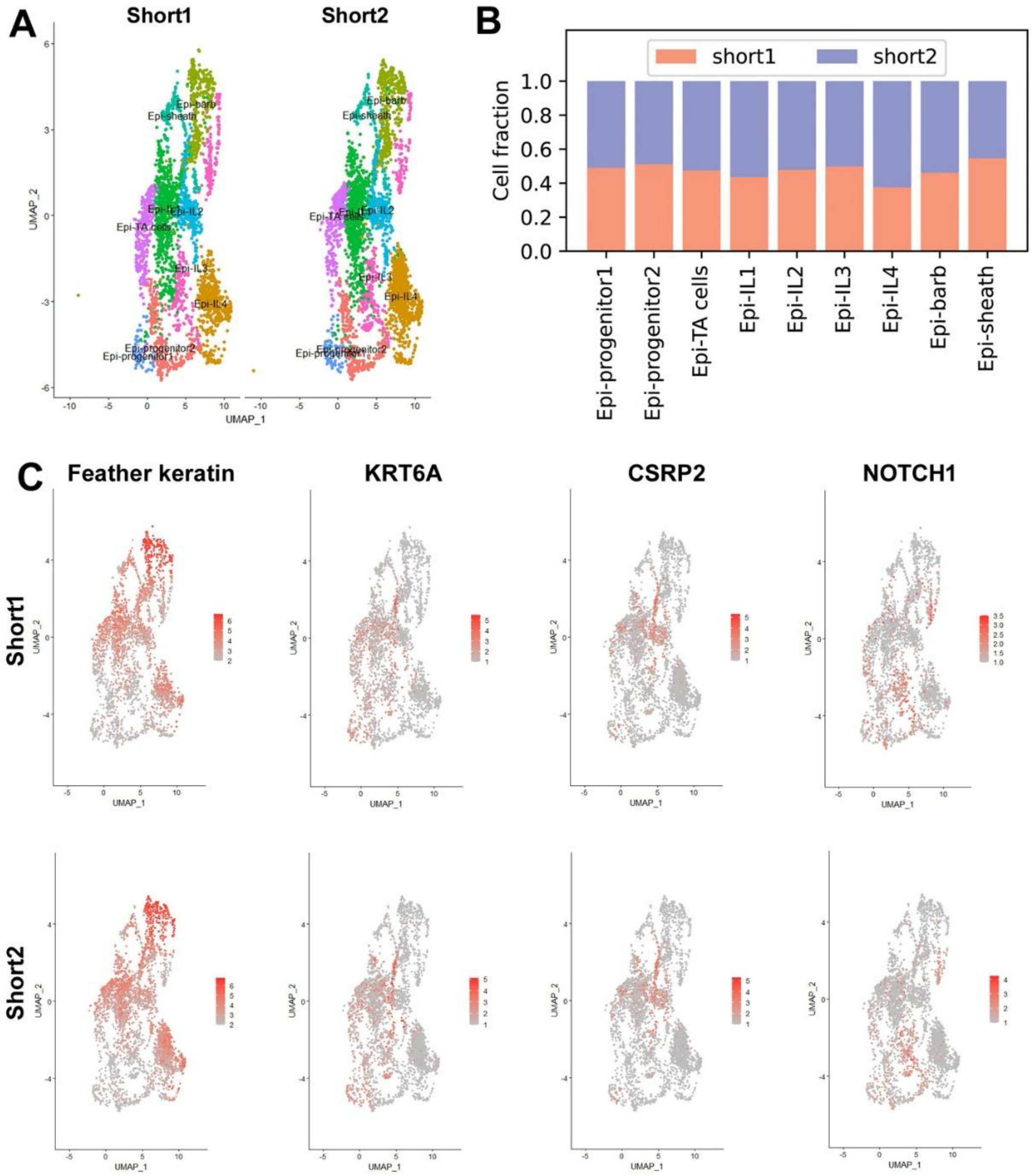

Figure S5. The two short feather scRNA-seq replicates show very similar profiling.

A. Nine epidermal cell clusters are identified in two short feathers.

B. The cell fraction of two short feathers shows the similarity in most cell clusters.

C. Expression of major differential markers is similar in two short feather replicates.

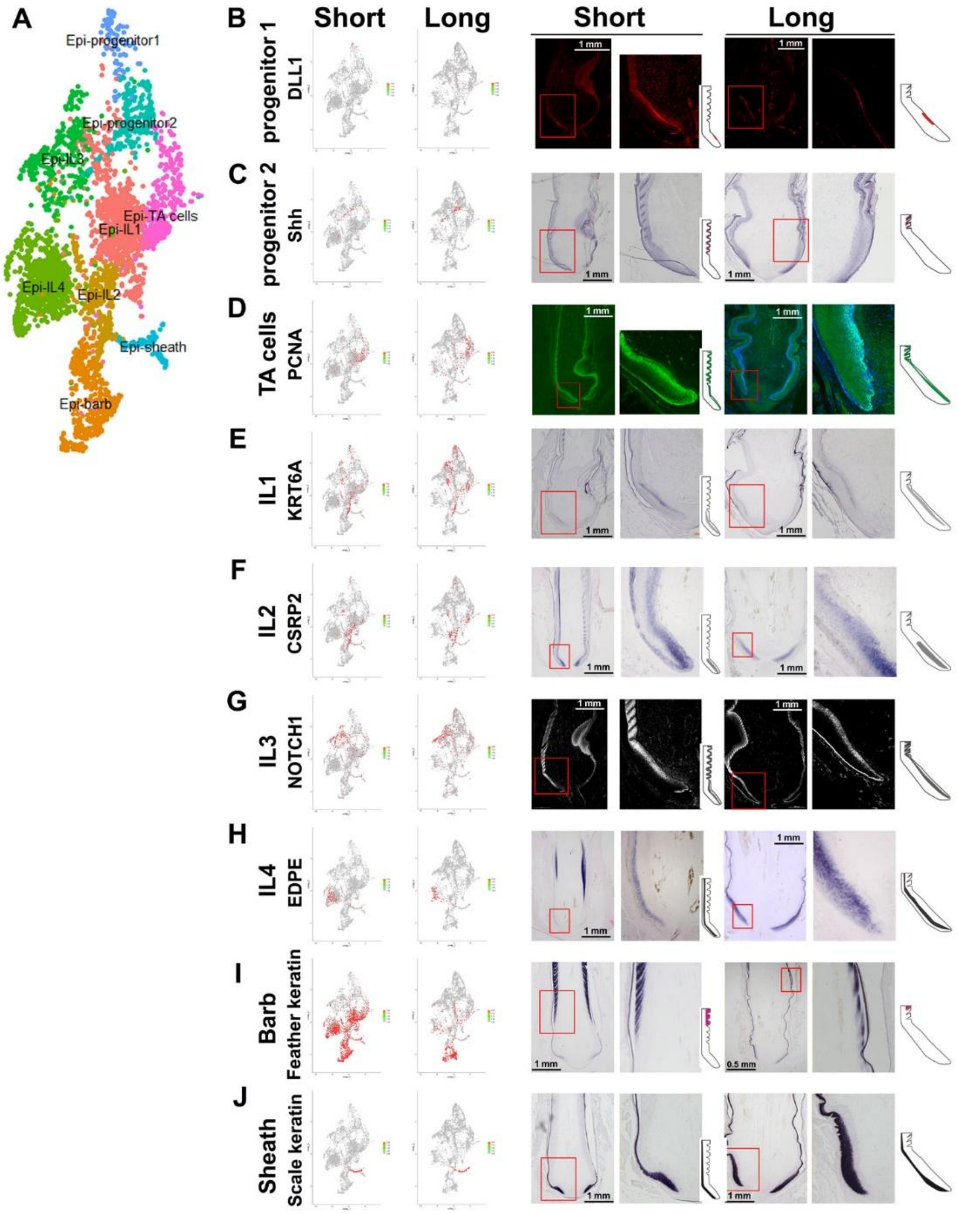

Figure S6. Epidermal cell clusters and localization

A. Epidermal cell clusters include two progenitor clusters, one TA cell cluster, four intermediate layer cell clusters and two terminal differentiation cell clusters.

B-J. Cell cluster distribution of epidermal marker genes. First and second columns, the expression in scRNA-seq cell clusters of short and long feathers, respectively. The third column shows marker gene locations in short or long feather follicles. B and G, RNAscope. D, immunostaining. C, E-F, H-J, in situ hybridization.

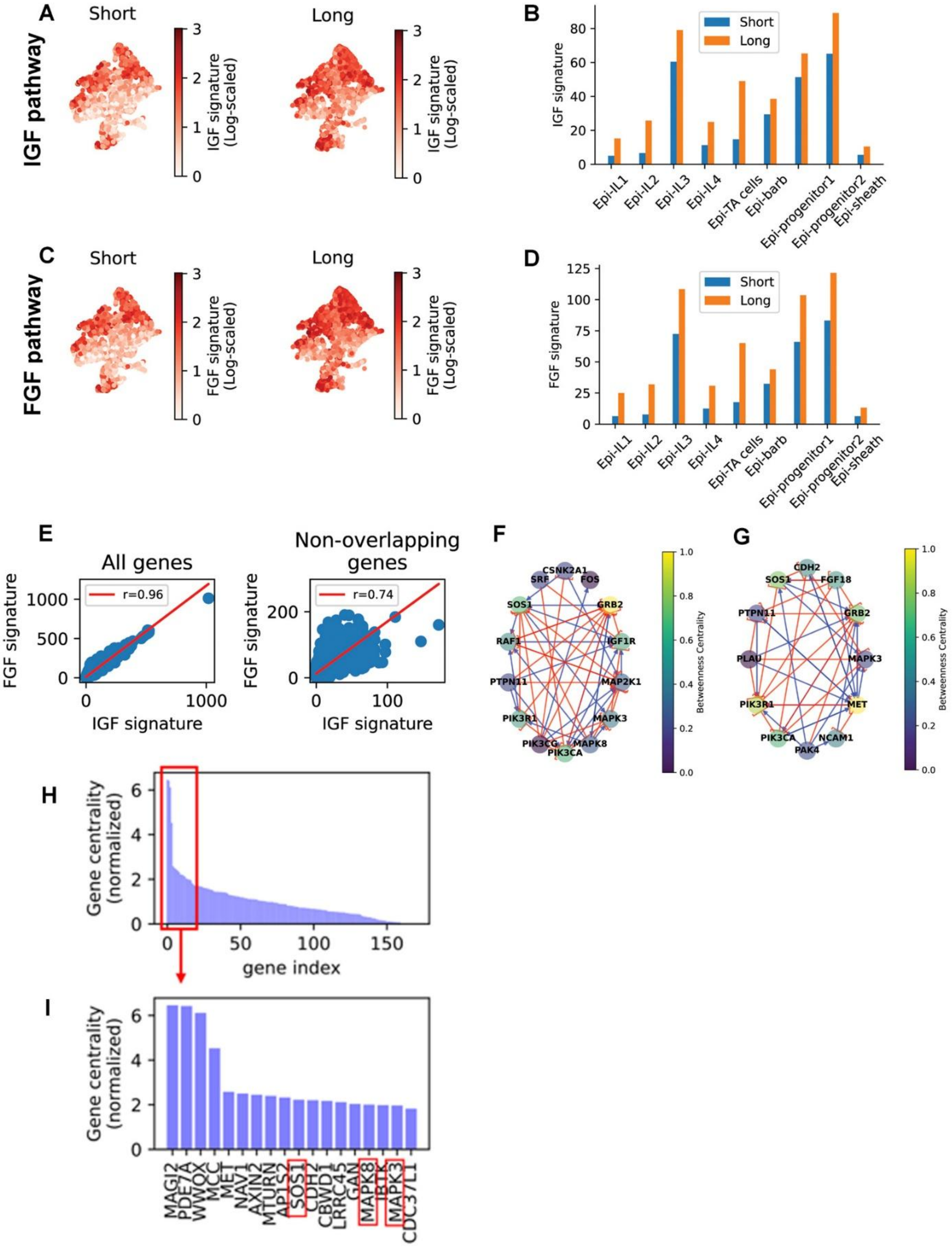
A
B
C
D
E
F
G
H
I
IGF pathway
FGF pathway
Short
Long
IGF signature (Log-scaled)
FGF signature (Log-scaled)
IGF signature
FGF signature
Epi-IL1
Epi-IL2
Epi-IL3
Epi-IL4
Epi-TA cells
Epi-barb
Epi-progenitor1
Epi-progenitor2
Epi-sheath
All genes
Non-overlapping genes
r=0.96
r=0.74
Betweenness Centrality
CSNK2A1
SRF
FOS
SOS1
GRB2
RAF1
IGF1R
PTPN11
MAP2K1
PIK3R1
MAPK3
PIK3CG
MAPK8
PIK3CA
CDH2
FGF18
PLAU
MET
NCAM1
PAK4
Gene centrality (normalized)
gene index
MAGI2
PDE7A
WWOX
MCC
MET
NAV1
AXIN2
MTURN
AP1S2
SOS1
CDH2
CBWD1
LRRC45
GAN
MAPK8
BTK
MAPK3
CDC37L1

Figure S7. Analysis of IGF and FGF signaling from scRNA-seq

A. Expression of the IGF gene signatures in the short and long feather epidermal cells.

B. Average expression of the IGF signature in each epidermal cluster for the short (blue) and long (orange) feather epidermal cells.

C. Expression of the FGF gene signatures in the short and long feather epidermal cells.

D. Average expression of the FGF signature in each epidermal cluster for the short (blue) and long (orange) feather epidermal cells.

E. The correlation of IGF and FGF pathways in analyzed cells.

F. A core IGF regulatory network inferred in the long feather epidermal cells.

G. A core FGF regulatory network inferred in the long feather epidermal cells.

H. The genes in the long feather epidermis ranked based on their betweenness centrality, whereby a high centrality score implies that the gene is more involved in gene regulation.

I. The top 18 genes in the long feather epidermal cells based on betweenness centrality. Red boxes highlight 3 highly connected genes that are part of both the IGF and FGF pathways (SOS1, MAPK8, MAPK3).

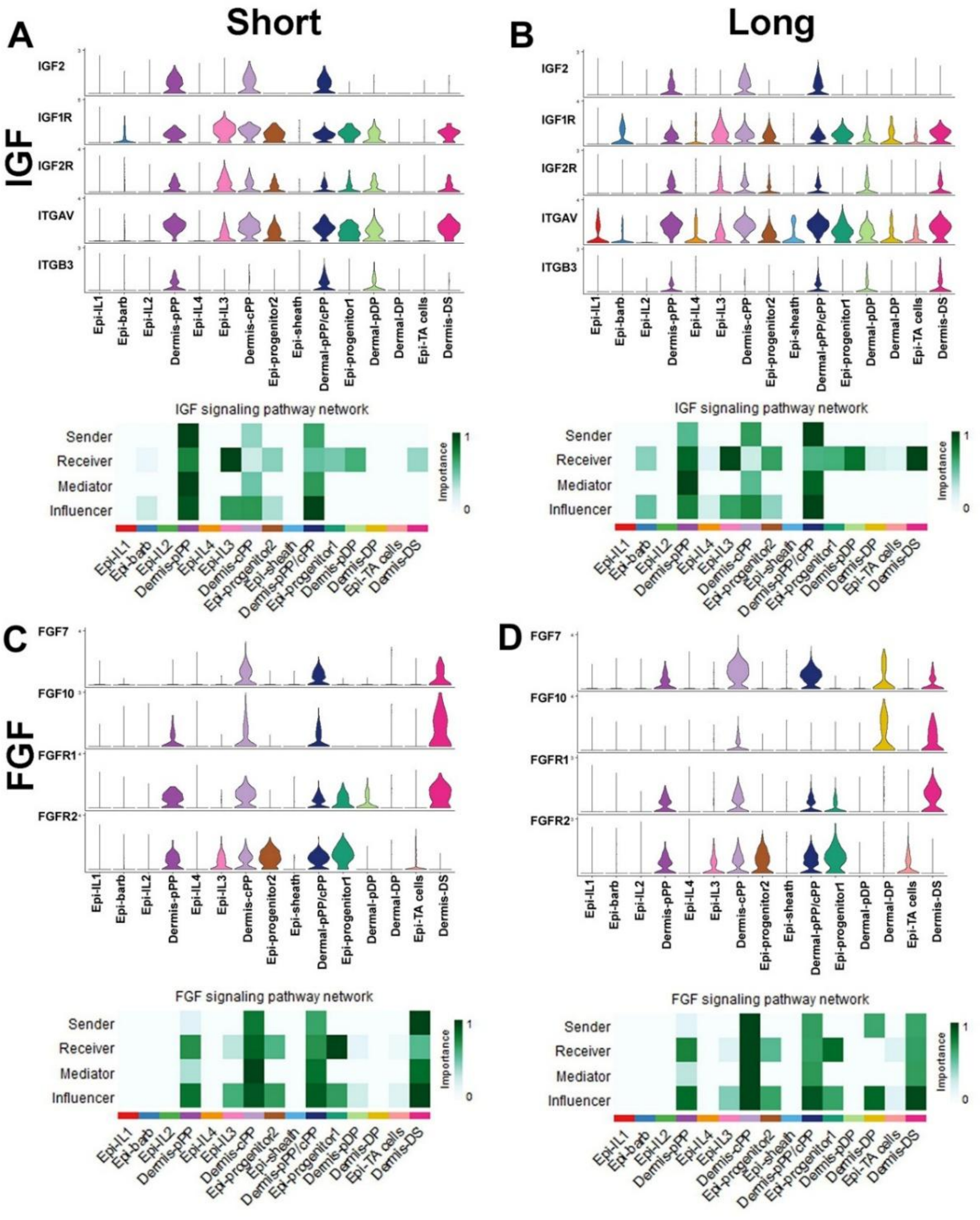

Figure S8. Comparative analysis of cell cluster signaling roles in the IGF and FGF signaling between short and long feathers.

A-B, IGF pathway. C-D, FGF pathway. A and C, short feathers. B and D, long feathers. In each panel, the top figure is the violin plot depicting the expression of each ligand and receptor involved in the pathway. The lower panel quantifies the signaling role of each cluster as sender, receiver, mediator or influencer of the pathway.

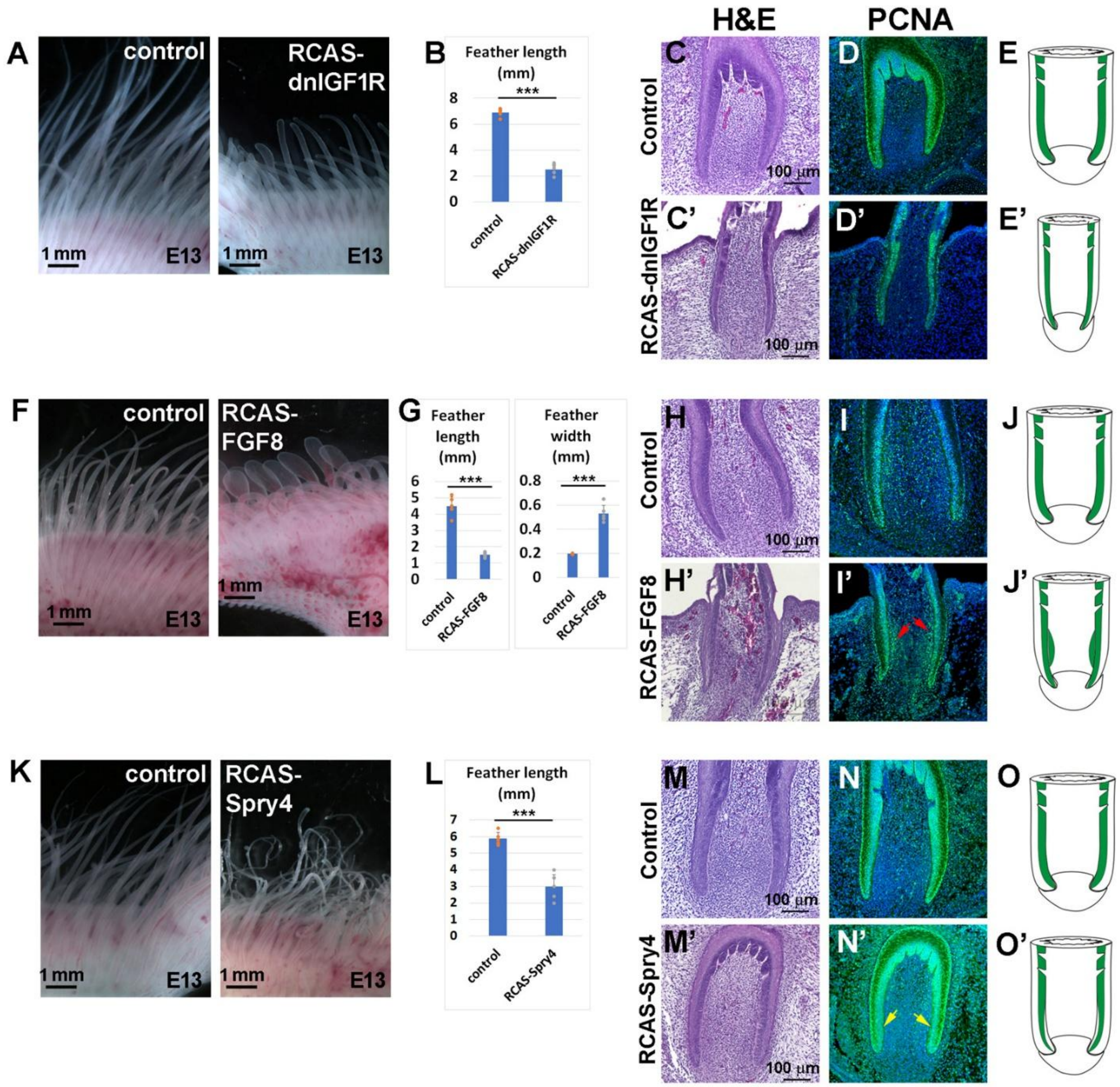

Figure S9. More IGF/FGF functional studies showing the role of IGF/FGF signaling in feather size control

A-E'. RCAS-dnIGF1R. F-J'. RCAS-FGF8. K-O'. RCAS-Spry4. Red arrows in I' indicate the ectopic proliferation zone in RCAS-FGF8 treated sample. Yellow arrows in N' indicate the reduced PCNA domain in the follicle base of RCAS-Spry4 treated sample. For panels B, G and L, five flight feather filaments from the right (experiment) and left (control) wings were measured. *** $P<0.001$.

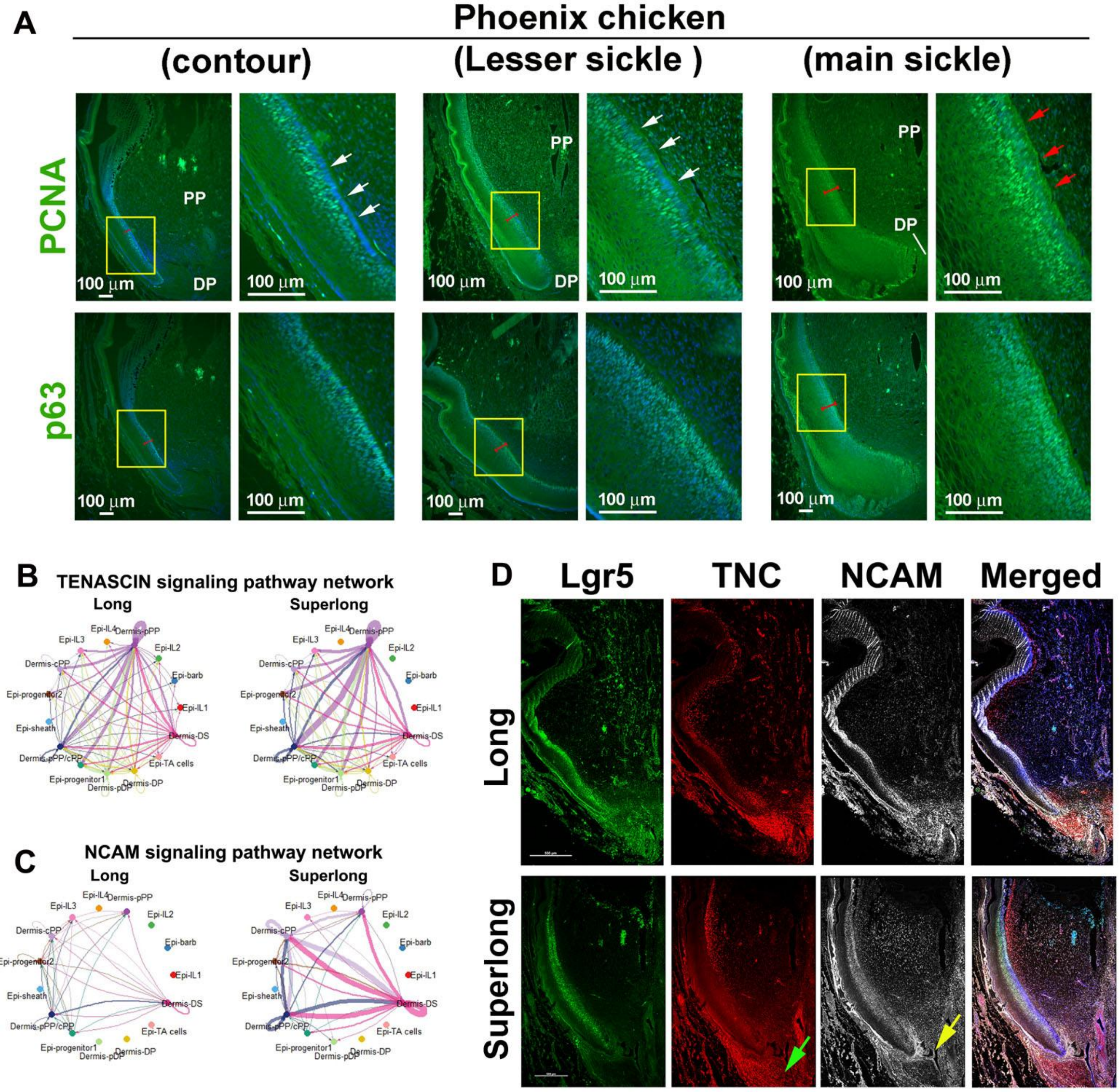

Figure S10. More analysis of Phoenix chicken feathers

A. PCNA and p63 staining in Phoenix chicken feathers. Note the contour and lesser sickle feather follicles have a PCNA negative zone (white arrows) but it is absent in the main sickle feather (red arrows). Red bar indicates the width of the expression domain.

B-C. Example of increased cell-cell interaction in superlong Phoenix sickle feathers, compared to White leghorn chicken sickle feathers. B, TENASCIN signaling pathway. C, NCAM signaling pathway.

D. RNAscope analysis showing the increased TENASCIN-C (TNC) (green arrow) and NCAM (yellow arrow) in the Phoenix sickle feathers.

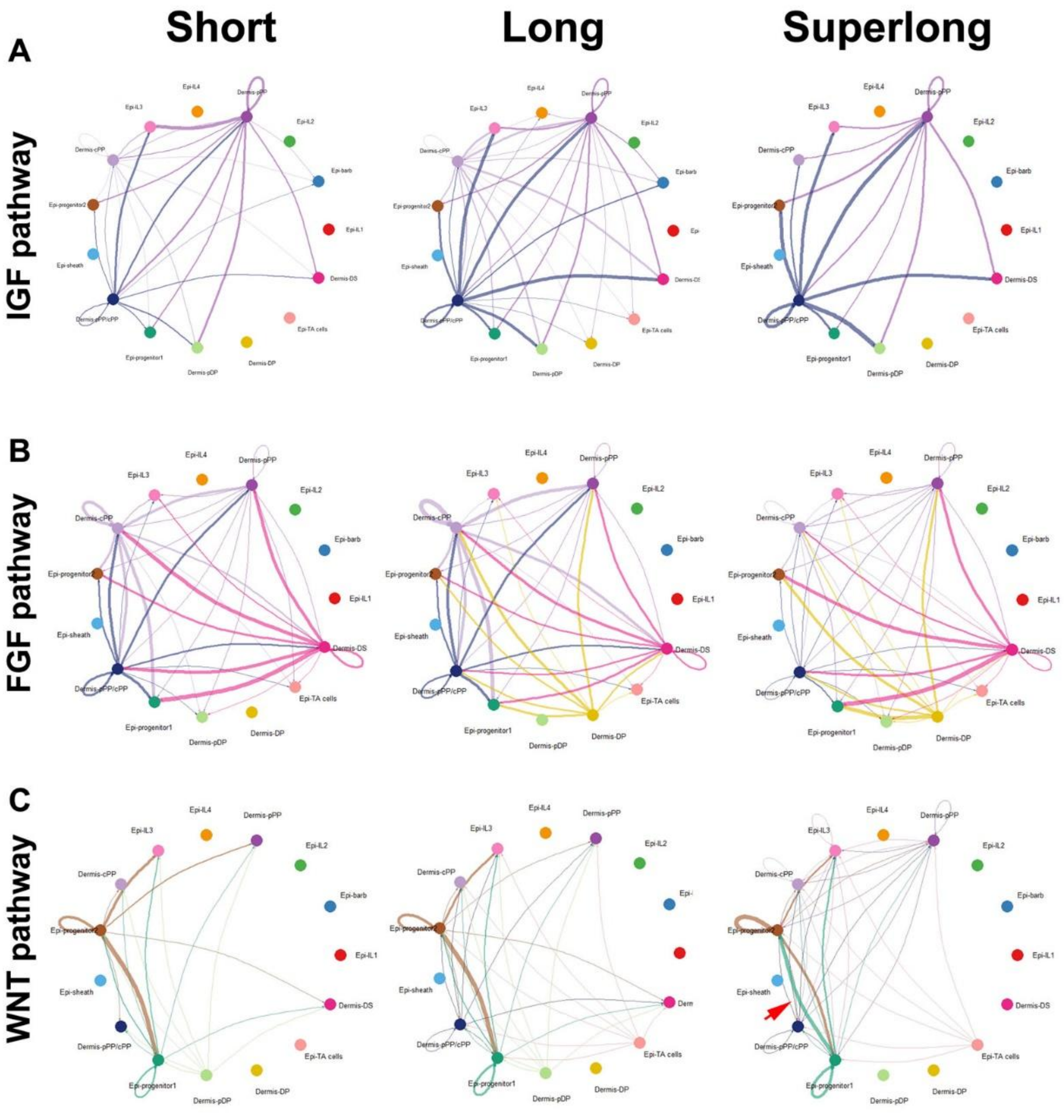

Figure S11. Comparative Cellchat analysis indicates that the superlong feather has more WNT signaling interactions between progenitor 1 and 2 clusters

A-C. Different pathways. A, IGF pathway. B, FGF pathway. C, Wnt pathway. Columns 1-3. Different feathers. Left and middle, short feather and long feather from White leghorn chicken. Right, superlong feather from Phoenix chicken. Note that superlong feathers do not have more interactions in IGF and FGF pathways, compared to the long feather. However, superlong feathers have more WNT signaling interactions between progenitor 1 and 2 (red arrow).

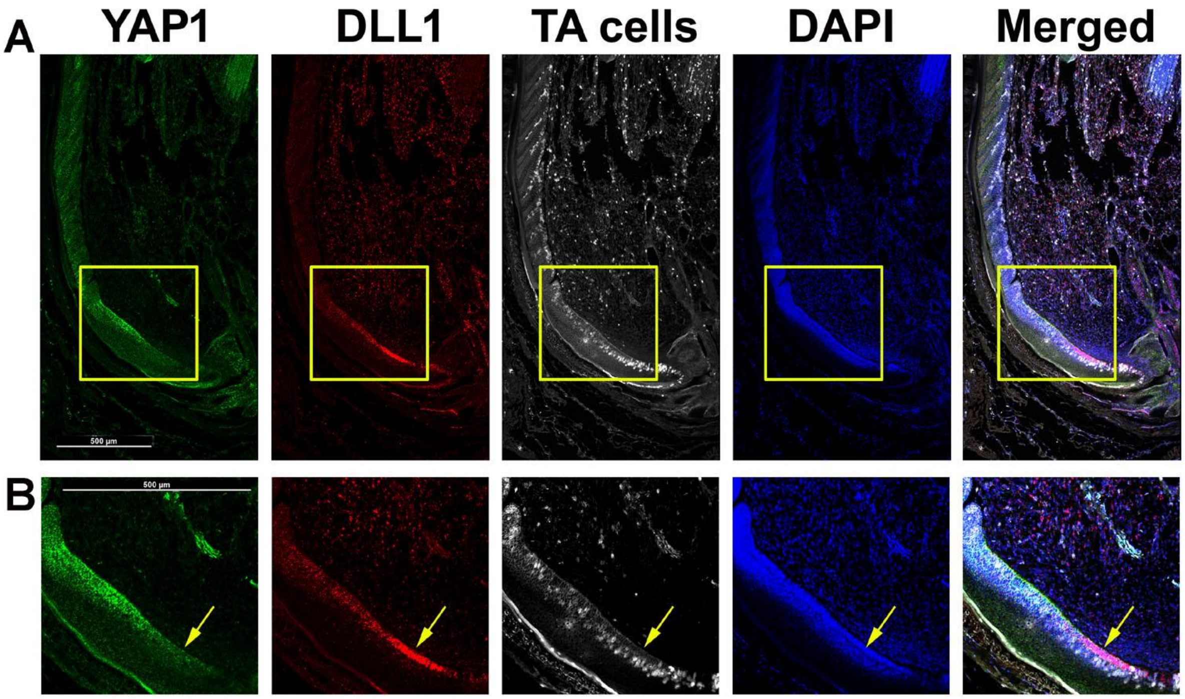

Figure S12. The expression of YAP1 / DLL1 and the distribution of TA cells

A-B. RNAscope analysis of YAP1/DLL1 followed by CldU staining (TA cells) show DLL1 is absent in the TA cell zone in White leghorn contour feathers. Note the DLL1 expression is in the collar bulge and is surrounded by TA cells.

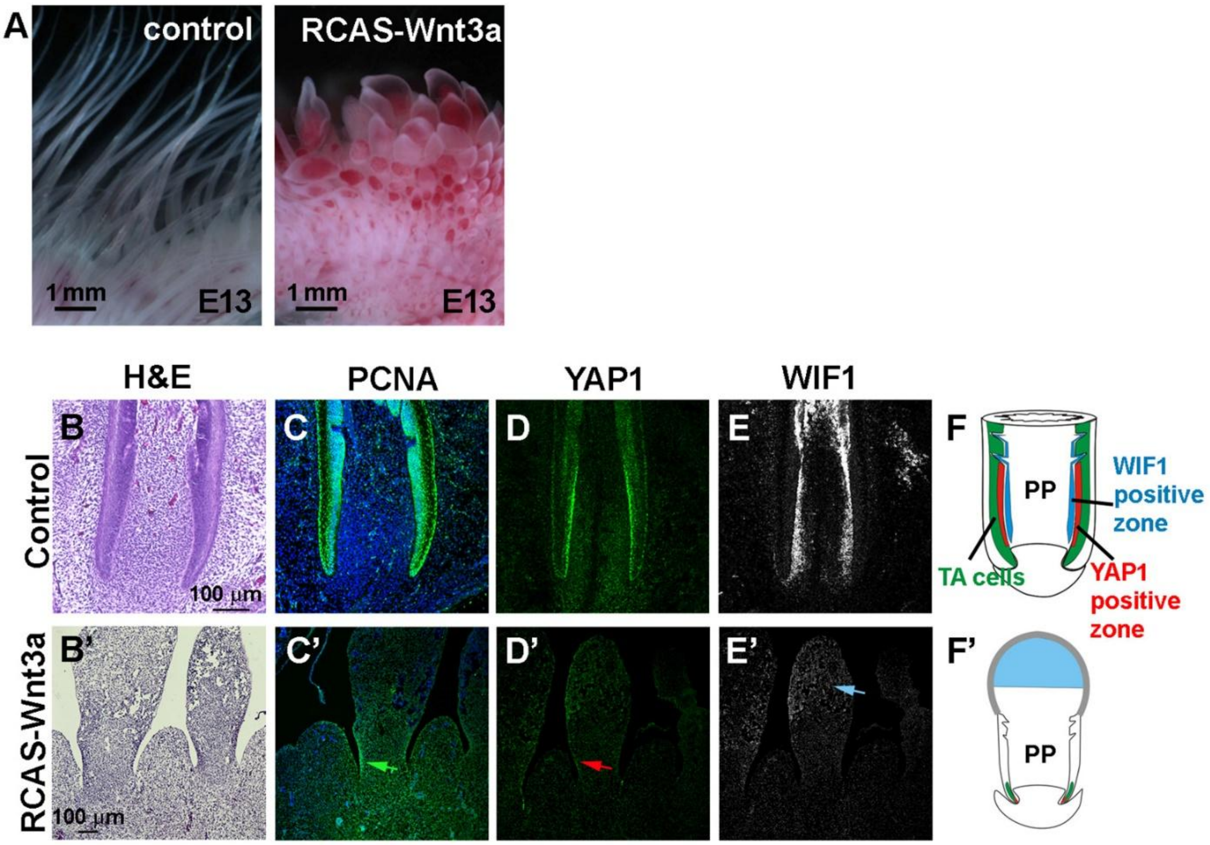

Figure S13. RCAS-Wnt3a treated sample shows the short and fat feather filament.

A. Bright view. B-E. Control sample. B'-E'. RCAS-Wnt3a treated sample. Green arrow in C' indicates the reduced PCNA expression zone is restricted to the base of the follicle. The red arrow in D' indicates the reduced YAP expression zone. The blue arrow in E' indicates the faint expression of WIF1 in the pulp of the upper follicle.

Table S1. Differential gene expression of epidermal progenitor cells

Table S2. Gene ontology analysis from different stage epidermal progenitor cells

Table S3. Marker genes of 30 cell clusters from feather follicle scRNA-seq

Table S4. Marker genes after re-clustering epidermal and dermal clusters

Table S5. Sub-clustering epidermal progenitor cell cluster

Table S6. Comparison of YAP/DLL1/NOTCH1 in epidermal cells among different feathers

	Pre-ramogenic zone (progenitor 2)	Collar bulge (progenitor 1)	Papilla ectoderm	Dermal sheath
Short feather				
YAP1	++	-	-	-
DLL1	-	+	-	-
NOTCH1	+++	++	+	+
Long feather				
YAP1	++	±	±	±
DLL1	-	++	-	-
NOTCH1	++	++	+	+
Phoenix chicken superlong feather				
YAP1	++	±	±	++
DLL1	-	+++	+++	+++
NOTCH1	++	++	++	++
